# Molecular Characterization Revealed the Role of Thaumatin-Like Proteins of Bread Wheat in Stress Response

**DOI:** 10.3389/fpls.2021.807448

**Published:** 2022-01-11

**Authors:** Alok Sharma, Himanshu Sharma, Ruchika Rajput, Ashutosh Pandey, Santosh Kumar Upadhyay

**Affiliations:** ^1^Department of Botany, Panjab University, Chandigarh, India; ^2^Department of Biotechnology, I.K. Gujral Punjab Technical University, Jalandhar, India; ^3^National Institute of Plant Genome Research, New Delhi, India

**Keywords:** abiotic stress, biotic stress, cereals, thaumatin-like proteins, *TaTLP2-B*

## Abstract

Thaumatin-like proteins (TLPs) are related to pathogenesis-related-5 (PR-5) family and involved in stress response. Herein, a total of 93 *TLP* genes were identified in the genome of *Triticum aestivum.* Further, we identified 26, 27, 39, and 37 *TLP* genes in the *Brachypodium distachyon*, *Oryza sativa*, *Sorghum bicolor*, and *Zea mays* genomes for comparative characterization, respectively. They could be grouped into small and long TLPs with conserved thaumatin signature motif. Tightly clustered genes exhibited conserved gene and protein structure. The physicochemical analyses suggested significant differences between small and long TLPs. Evolutionary analyses suggested the role of duplication events and purifying selection in the expansion of the TLP gene family. Expression analyses revealed the possible roles of *TLPs* in plant development and abiotic and fungal stress response. Recombinant expression of *TaTLP2-B* in *Saccharomyces cerevisiae* provided significant tolerance against cold, heat, osmotic, and salt stresses. The results depicted the importance of TLPs in cereal crops that would be highly useful in future crop improvement programs.

## Introduction

The growth and development of plants have always been affected by various abiotic and biotic stress conditions. In response to these stress conditions, plants produce numerous molecules including pathogenesis-related (PR) proteins (Kombrink and Somssich, 1997). PR proteins belong to a superfamily of defense-related proteins consisting of PR1-PR17 protein families that have been classified based on amino acid sequences, serological reaction, enzymatic activity, and so on (Christensen et al., 2002; van Loon et al., 2006).

Thaumatin-like proteins (TLPs) are related to the PR5 family of the PR superfamily (van Loon et al., 2006). These are called TLPs due to their significant similarity with a ∼23 kDa sweet-tasting protein known as thaumatin (Velazhahan et al., 1999). Thaumatin protein was first isolated from an African shrub known as *Thaumatococcus danielli* (van der Wel and Loeve, 1972). Over the period, TLPs have been reported in a diverse group of organisms including insects, nematodes, fungi, and plants (Sakamoto et al., 2006; Shatters et al., 2006; Liu et al., 2010a; Cao et al., 2016). In plants, TLPs are reported from algae to angiosperms (Liu et al., 2010a,b; Petre et al., 2011; Cao et al., 2016).

Based on the molecular weight (MW), TLP proteins are grouped as long (L-type) and small (S-type) TLPs, having the MW of ∼21–26 kDa and ∼16–17 kDa, respectively. Long TLPs have been identified from each group of plants, while small TLPs are confined to only gymnospermous and monocot plants (Liu et al., 2010a,b; Petre et al., 2011; Cao et al., 2016). A total of 16 and 10 conserved cysteine residues, forming eight and five disulfide linkages, are known to be present in long and small TLPs, respectively. These disulfide bonds provide resistance against extreme pH, heat, and protease degradation (Fierens et al., 2009; Liu et al., 2010a). Each TLP consists of a conserved REDDD motif and a thaumatin signature motif G-X-[GF]-X-C-X-T- [GA]-D-C-X- (1,2)-G-X-(2,3)-C. The REDDD motif is involved in receptor binding for its antifungal action (Liu et al., 2010a). Numerous studies reported that the overexpression of *TLPs* provides significant resistance against various fungi in both dicot and monocot plants (Datta et al., 1999; Rajam et al., 2007; Wang et al., 2010; Cui et al., 2021). The transgenic *Arabidopsis thaliana* with an overexpressing *PR5* gene (*ObTLP1*) of *Ocimum basilicum* showed enhanced tolerance against *Sclerotinia sclerotiorum* and *Botrytis cinerea* (Misra et al., 2016). The improved resistance of transgenic wheat against common root rot and leaf rust has been observed in *TaTLP1*-overexpressing transgenic lines (Cui et al., 2021). The mechanism of action of TLPs in fungal resistance is ambiguous; however, it has been presumed that they work by degradation and permeabilization of the fungal cell walls (Abad et al., 1996; Liu et al., 2010a). Besides, TLPs are also known to be involved in antifreeze action and abiotic stress resistance in plants (Zhang and Shih, 2007; Li Z. et al., 2020). The overexpression of *GhTLP19* in transgenic *A. thaliana* facilitated the plant with improved resistance against drought stress (Li Z. et al., 2020). Further, they are also reported to be involved in various development processes, for instance, flowering, fruit ripening, and seed germination (Neale et al., 1990; Salzman et al., 1998; Seo et al., 2008). Moreover, the upregulation of *TLPs* in response to ethylene, jasmonic acid, and salicylic acid treatment depicted their involvement in the hormonal signaling cascade (Yan et al., 2017; Li X. et al., 2020).

Despite utmost importance, a detailed characterization of TLPs is still limited in various cereal crops. Therefore, the current study aims at an inclusive characterization of TLPs in bread wheat, an important cereal crop. Besides, we have also performed a comparative analysis of various features of TLPs in other cereals including *B. distachyon*, *O. sativa*, *S. bicolor*, and *Z. mays*. Chromosomal distribution, phylogeny, physicochemical properties, gene duplication events (DEs), *cis*-regulatory elements, and gene and protein structural analyses were performed. To decipher their roles in plant development and under abiotic and biotic stresses, their expression analysis was performed using high throughput RNA-seq data. The expression of eight selected *TaTLP* genes was validated using qRT-PCR. An abiotic stress-responsive gene, *TaTLP2-B*, of *Triticum aestivum* was cloned and used for functional characterization in *Saccharomyces cerevisiae* (yeast) cells. Recombinant expression of TaTLP2-B provided significant tolerance to the yeast in spot assay against various abiotic stresses. The study will increase the knowledge about numerous TLP proteins and provide the base for the functional characterization of identified genes in future studies.

## Materials and Methods

### Plant Materials, Growth Conditions, and Stress Treatments

Surface sterilization of bread wheat seeds (cv. Chinese Spring) was done with the solution having 1.2% sodium hypochlorite in 10% ethanol. Thereafter, the seeds were washed carefully with the double autoclaved water. The sterilized seeds were kept overnight on moist Whatman filter papers at 4°C for stratification and were further kept at room temperature for germination. The seedlings were then transferred to fresh phytajars and were allowed to grow in plant growth chambers under 60% relative humidity, 22°C temperature, and a 16 and 8 hours (h) light and dark period, respectively (Lei et al., 2021). For the stress treatments, the 7-days-old seedlings were kept under heat (40°C), osmotic [20% Polyethylene glycol (PEG)], and combined heat and osmotic stress treatments (40°C and 20% PEG) for 6, 12, 24, and 48 h. Similarly, for salinity stress, 7-days-old seedlings were subjected to 150 mM NaCl treatment for 6, 12, 24, and 48 h. However, for the control plants, the seedlings grown in normal conditions were used. The plant samples including roots and shoots were collected at the interval of 6, 12, 24, and 48 h of stress treatments and stored at –80°C till further experiments. These stress conditions were selected based on the earlier reports. Further, the RNA seq data of *T. aestivum* used for the *in silico* expression analyses were generated under the same conditions (Liu et al., 2015; Zhang et al., 2016).

### Identification and Nomenclature of the Thaumatin-Like Protein Genes

Extensive BLAST searches were used to identify the TLP proteins in five different cereals including *B. distachyon*, *O. sativa*, *S. bicolor*, *T. aestivum*, and *Z. mays*. Arabidopsis TLP sequences were used as a query against the protein model sequences of each crop downloaded from the Ensembl Plants (Petre et al., 2011; Sharma A. et al., 2020). The presence of the thaumatin (PF00314) domain was analyzed using the Hidden Markov Model (HMM) and Pfam BLAST searches at an *e*-value 10^––10^. Identified TLP sequences were further subjected to the NCBI Conserved Domain Database (CDD) BLAST to further confirm the presence of the thaumatin domain. The TLP proteins, having a complete thaumatin family signature, were selected for further analysis. The identified *TLPs* were named as per their sequence of occurrence at various chromosomes in each crop, except *T. aestivum*. The international rules for gene symbolization of *T. aestivum*^1^ were followed for the nomenclature of *TaTLPs*.

### Chromosomal Localization and Duplication Events

Ensembl Plant^2^ was used for gathering the chromosomal and sub-genomic location of *TLPs* of all five crops. The homeologous *TLP* genes in *T. aestivum* were identified based on ≥ 90% sequence similarity and their occurrence at the related chromosomes. The MapInspect software was used for the graphical representation of *TLP* genes on their respective chromosomes.^3^ DEs were identified using a bidirectional blast hit approach with sequence similarity of ≥ 80%, while tandem and segmental DEs were segregated based on their distance and occurrence at respective chromosomes as per the previous studies (Shumayla et al., 2019).

### Multiple Sequence Alignments, Phylogeny, and Structural Analysis

The Muscle and Multalin tools were used for the multiple sequence alignments of all the TLP*s* with a known thaumatin protein (P02883.2| THM1_THADA) to find the conserved residues (Corpet, 1988; Edgar, 2004). The phylogenetic tree was constructed by the maximum likelihood method with 1,000 bootstraps using the MEGA X software (Kumar et al., 2018).

### Ka/Ks and Tajima’s Relativity Test

The alignment of the protein and nucleotide sequences of the paralogous gene pairs was done using the ClustalOmega server.^4^ The synonymous substitution per synonymous site (Ks), the non-synonymous substitution per non-synonymous site (Ka), and their ratio (Ka/Ks) were calculated using the PAL2NAL program (Suyama et al., 2006; Nagaraju et al., 2020). The calculation of the divergence time of each pair of duplicated genes was done using the formula T = Ks/2r, where T represents the divergence time and r represents the divergence rate. The divergence rate was assumed to be 6.5 × 10^–9^ for cereals (Gaut et al., 1996). The Tajima’s relativity test was performed to find out the evolutionary rate between paralogous genes (Tajima, 1993).

### Gene Structure Organization

The genomic and coding DNA sequence (CDS) of identified *TLP* genes were used for the analysis of gene structure in terms of exon-intron organization and intron phases using the GSDS 2.0 server as done in earlier studies (Hu et al., 2015; Sharma H. et al., 2020).

### Physicochemical Properties of the Thaumatin-Like Proteins

Various physicochemical characteristics such as peptide length, MW, and isoelectric point (pI) were analyzed using the ExPasy tool, which were further confirmed from the Ensembl plants and Sequence Manipulation Suite (Stothard, 2000; Gasteiger et al., 2005; Nussbaumer et al., 2013). Tools including CELLO v.2.5, ngLOC, ProtComp9, and WoLF PSORT were used for the prediction of subcellular localization of TLP proteins (Emanuelsson et al., 2000; Yu et al., 2006; Horton et al., 2007; King and Guda, 2007). Tools such as Phobius and DAS-TMfilter were used for the prediction of transmembrane regions (Cserzo et al., 2004; Käll et al., 2004). The signal peptides were detected using the tools Phobius and SignalP (Käll et al., 2004; Petersen et al., 2011). SMART server was used for the domain analysis, whereas, motifs were analyzed using MEME v.4.11.4 (Bailey et al., 2009; Letunic et al., 2015).

### *Cis*-Regulatory Element Analysis

For *cis*-regulatory elements analysis, 1.5 kb upstream genomic sequences from the initiation codon were retrieved for each *TLP* gene. These promoter sequences were analyzed using the PLACE software (Higo et al., 1998). The identified *cis*-regulatory elements were categorized based on their functions.

### Expression Profiling Using RNA-Seq Data

Expression analysis in various tissues of each crop was done using the high throughput RNA-seq data retrieved from the Unité de Recherche Génomique Info (URGI) database^5^ and Expression ATLAS (Choulet et al., 2014; Pingault et al., 2015; Papatheodorou et al., 2018). In *T. aestivum*, the expression data generated in replicates for various tissue developmental stages, under biotic (fungal pathogen) and abiotic (heat, osmotic and salt) stress conditions in various studies, were used (Zhang et al., 2014, 2016; Liu et al., 2015). Data available for three developmental stages of root, stem, leaf, spike, and grain were used to analyze the tissue-specific expression in wheat. Further, the RNA-seq data (PRJNA243835) available in triplicates after the 24, 48, and 72 h of inoculation of *Blumeria graminis* f. sp. *tritici* (Bgt) and *Puccinia striiformis* f. sp. *tritici* (Pst) in 7-days-old seedlings were used for the *TaTLP* expression analysis under biotic stress (Zhang et al., 2014). Under abiotic stress conditions, duplicate RNA-seq data (SRP045409) for 1 and 6 h of treatments of heat (40°C), osmotic (20% PEG 6000), and a combination of both heat and osmotic stresses were used to study the *TaTLP* expression (Liu et al., 2015). Moreover, Liu et al. (2015) used the term drought stress for PEG treatment, but in the current study, the term osmotic stress has been used for PEG treatment because it actually causes osmotic stress that leads to water stress. Root RNA-seq data (SRP062745) available in triplicates for the treatment of 150 mM NaCl at 6, 12, 24, and 48 h were used to analyze the expression of *TaTLPs* under salt stress (Zhang et al., 2016). Trinity package was used to calculate the expression value in terms of fragments per kilobase of exon per million mapped fragments (FPKM) value (Haas et al., 2013). Hierarchical Clustering Explorer 3.5 was used to generate heat maps of differentially expressed genes, which were clustered using the Euclidean distance method (Seo et al., 2006).

### RNA Isolation, cDNA Synthesis, and qRT-PCR

The 7-days-old seedlings treated with various abiotic stresses were harvested after the 6, 12, 24, and 48 h of stress treatments. The root and shoot tissues of these seedlings were used for the total RNA isolation. The total RNA of each sample was isolated using the Spectrum™ Plant Total RNA kit (Sigma, United States). The seedlings (shoot and root) grown under normal conditions were used as a control. The TURBO DNA-free ™ Kit (Invitrogen, United States) was used to remove the genomic DNA contamination. The qualitative and quantitative analysis of RNA were done using agarose gel electrophoresis and NanoDrop quantification, respectively. The Superscript III First-Strand Synthesis Super-mix (Invitrogen, United States) was used to synthesize the cDNA from one microgram of total RNA. A real-time qRT-PCR was performed with the gene-specific primers of selected genes using SYBR Green at the 7900 HT Fast Real-Time PCR System (Applied Biosystems) following the method established in our laboratory (Shumayla et al., 2019). The fold expression change was calculated using the delta-delta CT method (2^–ΔΔ*CT*^) using the expression of ADP-ribosylation factor (*TaARF*) as an internal control as reported in earlier studies (Livak and Schmittgen, 2001; Shumayla et al., 2019; Tyagi et al., 2021). All the experiments were carried out in three biological replicates and expressed as mean ± SD. A significant difference between the control and treatments was examined by using the two-tailed student’s *t*-test.

### Cloning and Functional Characterization

The full-length open reading frame (ORF) of the *TaTLP2-B* gene was amplified from cDNA using the *TaTLP2-B* forward (5′ GTAATGGCTCTTCTTCCTCCTCTGCTTCTG 3′) and *TaTLP2-B* reverse (5′ AATCTGGGCCACACGATCGCCCC 3′) primers. The amplified DNA was cloned into the pJET1.2 cloning vector and sequenced. *TaTLP2-B* gene was re-amplified from the confirmed clone using the same primers and ligated into the pYES2.1/V5-His-Topo vector (Invitrogen, United States). The recombinant plasmid (pYES2.1-*TaTLP2-B*) was transformed in *S. cerevisiae* (W303) (yeast) cells for further characterization. For control, *lacz* gene containing pYES2.1 vector (pYES2.1/V5-His/lacZ) was used during the studies. Spot assays using recombinant yeast cells were performed under various abiotic stress conditions. Recombinant yeast cells were grown overnight in SD/-ura (with 2% dextrose) medium at 30°C and 200 rpm as primary culture and further inoculated for secondary culture (1:100 dilutions). As soon as OD_600_ reached 0.4, 2%, galactose was added to induce the expression of recombinant protein in yeast and kept at 30°C and 200 rpm for 6 h. The OD_600_ was adjusted to 0.4 and an equal volume (500 μl) of each induced culture was further diluted in 10 ml medium. The diluted cultures were treated with heat (37 and 40°C), cold (4°C), osmotic (20 and 30% PEG), combined heat and osmotic (37°C, and 30% PEG), and salt (1 M NaCl) stresses for 24 h, separately. After the treatments, serial dilutions (10^0^, 10^–2^, 10^–4^, and 10^–6^) were prepared, 5 μl of each dilution was spotted on SD/-ura agar plates, and incubated at 30°C for 2–3 days as reported in earlier studies (Shumayla et al., 2019). All the experiments were performed in triplicates and the results were compared visually.

### Subcellular Localization of the TaTLP2-B

Subcellular localization of TaTLP2-B protein was analyzed using CaMV35S-driven C-terminal yellow fluorescent protein (YFP) fusion construct generated by Gateway LR-recombination into binary vector PEG101. Plasmids were then transformed into *Agrobacterium tumefaciens* GV3101 strain. The cells were resuspended in a freshly prepared infiltration medium (10 mM MgCl_2_, 10 mM MES/KOH, pH 5.7 and 150 μM acetosyringone). The resulting constructs were infiltrated onto the abaxial surface of *Nicotiana benthamiana* leaf and kept at 22°C for 48 h. The YFP fluorescence was observed using the Leica TCS SP8 (Leica Microsystems, Wetzlar, Germany) laser-scanning confocal microscope at 514–527 nm.

## Results

### Identification and Chromosomal Localization of the Thaumatin-Like Protein Genes

Numerous properties of TLPs ascribed to defense and development pathways and lack of such studies in numerous cereals lead us to perform a comprehensive analysis of the TLP family in five major cereals. An extensive BLAST search identified 26, 27, 39, 93, and 37 *TLP* genes in *B*. *distachyon, O*. *sativa, S*. *bicolor, T*. *aestivum*, and *Z*. *mays*, respectively. The genes lacking or having incomplete thaumatin signature motifs were excluded from the study. Four *TLPs* were identified as small *TLPs* (sTLPs) in each *B. distachyon, O. sativa*, and *Z. mays*, while 10 and 20 *sTLPs* were detected in *S. bicolor* and *T. aestivum*, respectively (Supplementary File 1). In the case of *T. aestivum*, the identified *TLP* genes from the A, B, and D sub-genomes formed 32 homeologous groups based on their sequence homology (≥90%). All of the identified proteins exhibited a complete thaumatin signature motif (Supplementary Figure 1).

**FIGURE 1:**
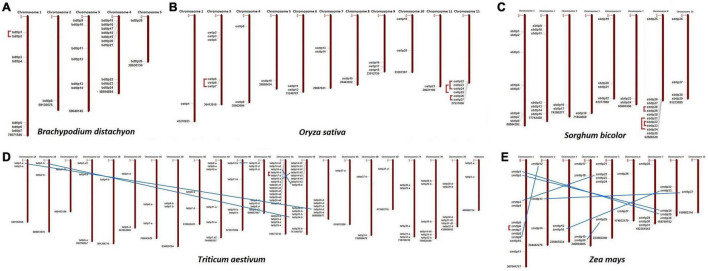
Chromosomal distribution and paralogous gene analysis of Thaumatin-like proteins *(TLPs)* in five cereal crops. Image depicts the scattered localization of *TLP* genes of **(A)** *B. distachyon*, **(B)** *O. sativa*, **(C)** *S. bicolor*, **(D)** *T. aestivum*, and **(E)** *Z. mays* at their respective chromosomes. Red lines and blue lines denote the tandem and segmental duplication events, respectively.

The chromosomal localization suggested the scattered distribution of the *TLP* genes at the majority of chromosomes in each crop (Figure 1). In *B. distachyon*, chromosome 4 consisted of a maximum of 10 *TLP* (both long and small) genes, while in the case of *O. sativa, S. bicolor, T. aestivum*, and *Z. mays*, the majority of genes (6, 11, 18, and 11) were localized on chromosomes 12, 8, 5A, and 1, respectively.

### Multiple Sequence Alignments

**FIGURE 2:**
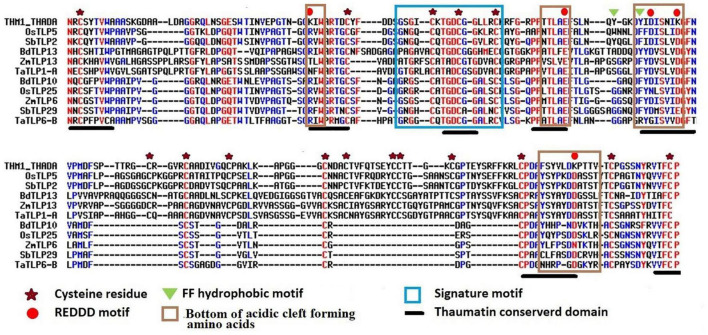
Multiple sequence alignment of selected long and small TLPs of *B. distachyon, O. sativa, S. bicolor, T. aestivum*, and *Z. mays* with a known thaumatin protein sequence. Figure shows the alignment of long (OsTLP5, SbTLP2, BdTLP13, ZmTLP13, and TaTLP1-A) and small (BdTLP10, OsTLP25, ZmTLP6, SbTLP29, and TaTLP6-B) TLPs with a known thaumatin (P02883.2| THM1_THADA) protein sequence. The characteristic thaumatin signature motif is shown in the sky blue box. All the conserved cysteine residues were marked with an asterisk. The REDDD motif was marked with a red circle. The FF hydrophobic motif and bottom of acidic cleft forming regions are marked with a green triangle and brown box, respectively. The conserved regions of the thaumatin domain are marked with a black line.

Thaumatin-like proteins consist of various characteristic and conserved residues, which are important for their functional activities. Multiple sequence alignments of TLP proteins with a known thaumatin protein (P02883.2| THM1_THADA) revealed the identification of 16 and 10 conserved cysteine residues in the majority of long and small TLP proteins of the studied cereal crops, respectively. Moreover, 7–15 cysteine residues had also been observed in a few TLPs of *O. sativa, S. bicolor, T. aestivum*, and *Z. mays*. The REDDD motif was also found conserved in the majority of TLP proteins. However, in certain TLP proteins, a few amino acid residues (AAs) were replaced by other AAs having either similar or different properties. For instance, Arginine (R) was replaced by Lysine (K) or Asparagine (N). This could be responsible for the differential evolvement of various characteristic features of TLPs during evolution. The thaumatin signature motif, which is a characteristic feature of TLPs (Liu et al., 2010a), was found to be highly conserved in all the identified TLP proteins. Some other regions like FF hydrophobic motif and bottom of acidic cleft forming amino acids were also found to be well conserved (Figure 2 and Supplementary Figure 1).

### Evolutionary Analyses

#### Phylogeny

**FIGURE 3:**
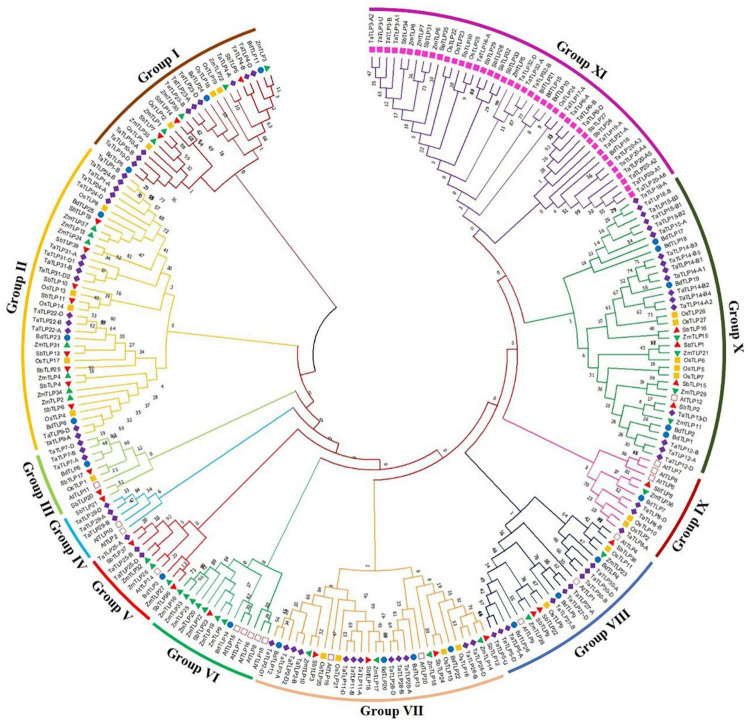
Phylogenetic tree analysis of *A. thaliana, B. distachyon, O. sativa. S, bicolor, T. aestivum, and Z. mays* using full-length peptide of TLPs. The figure shows the phylogenetic tree of TLPs, built by the maximum likelihood approach with 1000 bootstrap values using MEGA X software. The phylogenetic tree displays the clustering of TLPs of six plant species in I-XI different groups, each group is colored differently. The TLPs from each plant are marked with a different color.

To decipher the evolutionary relationship, a phylogenetic tree was built using the full-length TLP protein sequences of the five cereals and *A. thaliana*. These were clustered into 11 different clades based on their phylogenetic relatedness, named as groups I–XI (Figure 3). The highest number of TLPs was found in group XI, followed by group II and group X, while group IV was the smallest with only five genes. All the sTLPs were tightly clustered into group XI, which could be due to their smaller size. Further, the majority of groups consisted of TLPs from all the five cereals, except groups III, V, and VI that lacked members from one or more plant species. Besides, group IV comprised only three TaTLP and two AtTLP proteins. Besides, the homeologous TaTLPs of *T. aestivum* were tightly clustered in proximity.

#### Duplication Events Investigation

Duplication event analyses were carried out to understand their roles in the evolution and expansion of the TLP gene family in the studied crop species. A total of one, four, three, six, and eight DEs were predicted in *B. distachyon, O. sativa, S. bicolor*, *T. aestivum*, and *Z mays*, respectively (Figure 1 and Supplementary File 2). Only tandem DEs (TDEs) were found in the case of paralogous genes of *B. distachyon* (*BdTLP1-BdTLP2*), *O. sativa* (*OsTLP5-OsTLP7, OsTLP22-OsTLP24*, etc.), and *S. bicolor* (*SbTLP28-SbTLP29, SbTLP31-SbTLP34*, etc.). However, in the case of *T. aestivum*, five DEs (*TaTLP1-A-TaTLP24-D, TaTLP1-B-TaTLP24-B*, etc.) were segmental and one was TDE (*TaTLP16-A-TaTLP17-A*). In *Z. mays*, seven segmental (*ZmTLP1-ZmTLP35, ZmTLP2-ZmTLP34*, etc.) and one TDE (*ZmTLP6-ZmTLP7*) were found. All the duplicated gene pairs of each cereal crop were tightly clustered in proximity in the phylogenetic tree.

#### Ka/Ks and Tajima’s Relative Rate Test

Over the course of evolution, various evolutionary forces and natural pressures affected the duplicated genes (Ellegren, 2008). To understand the evolutionary divergence between the paralogous gene pairs, the Ka/Ks analysis was carried out. The Ka/Ks ratio of more than one suggests the positive (non-purifying) and less than one indicates negative (purifying) selection pressure. All the paralogous genes showed the Ka/Ks value lesser than one, which suggested the negative or purifying selection on duplicated *TLP* genes (Table 1). However, Ka/Ks analysis for *ZmTLP6* and *ZmTLP7* was not performed due to their 100% similarity. Additionally, the divergence time of DEs was also calculated using the Ks value and previously described methods (Huang et al., 2002; Sharma et al., 2014). The divergence time of duplicated genes was calculated as 83 million years ago (MYA) in *BdTLPs*, while the range varied from 52 to 108, 6 to 137, 63 to 99, and 1 to 63 MYA in *O. sativa, S. bicolor, T. aestivum* and *Z. mays*, respectively (Table 1). Tajima’s relative rate test found the insignificant χ^2^-value for all the duplicated gene pairs at *P* > 0.05 (Table 2). The *P*-value of more than 0.05 depicted their acceptance of the molecular evolutionary clock hypothesis (Tajima, 1993).

**TABLE 1:** The Ka/Ks ratio and divergence time of duplicated *TLP* gene pairs.

Gene A	Gene B	Ka	Ks	Ka/Ks	Selection pressure	T = Ks/2r
*BdTLPK1*	*BdTLPK2*	0.1102	1.0810	0.1020	Purifying	83.2
*OsTLP5*	*OsTLP7*	0.1014	0.6788	0.1494	Purifying	52.2
*OsTLP22*	*OsTLP24*	0.0697	0.4134	0.3190	Purifying	27.7
*OsTLP23*	*OsTLP25*	0.1149	0.3603	0.31646	Purifying	29.4
*OsTLP26*	*OsTLP27*	0.1569	1.3980	0.1122	Purifying	107.6
*SbTLP28*	*SbTLP29*	0.0911	0.6808	0.1338	Purifying	52.4
*SbTLP31*	*SbTLP34*	0.1388	1.7816	0.0779	Purifying	137
*SbTLP32*	*SbTLP33*	0.0055	0.0716	0.0771	Purifying	5.5
*TaTLP1-A*	*TaTLP24-D*	0.1224	0.8739	0.1401	Purifying	67.2
*TaTLP1-B*	*TaTLP24-B*	0.0937	0.8163	0.1148	Purifying	62.8
*TaTLP12-B*	*TaTLP13-D*	0.1193	1.1836	0.1008	Purifying	91
*TaTLP15-A*	*TaTLP18-B*	0.1368	0.9879	0.1384	Purifying	76
*TaTLP15-B2*	*TaTLP18-A*	0.1423	0.9582	0.1486	Purifying	73.7
*TaTLP16-A*	*TaTLP17-A*	0.1456	1.2825	0.1135	Purifying	98.7
*ZmTLP1*	*ZmTLP35*	0.0955	0.3929	0.2431	Purifying	30.2
*ZmTLP2*	*ZmTLP34*	0.1044	0.8149	0.1281	Purifying	62.7
*ZmTLP3*	*ZmTLP22*	0.0495	0.3036	0.1630	Purifying	23.4
*ZmTLP9*	*ZmTLP12*	0.0767	0.3783	0.2027	Purifying	29.1
*ZmTLP13*	*ZmTLP37*	0.0523	0.3200	0.1634	Purifying	24.7
*ZmTLP16*	*ZmTLP33*	0.0133	0.0141	0.9483	Purifying	1.1
*ZmTLP20*	*ZmTLP25*	0.0797	0.0889	0.8965	Purifying	6.9

*Ka, non-synonymous substitutions per non-synonymous site; Ks, synonymous substitutions per synonymous site; T, Divergence time.*

**TABLE 2:** Tajima’s relative rate test of paralogous genes.

Group A	Group B	Outgroup	Nt	Na	Nb	χ^2^	*P*
*BdTLP1*	*BdTLP2*	*BdTLP11*	403	47	42	0.28	0.59611
*OsTLP5*	*OsTLP7*	*OsTLP18*	401	28	29	0.02	0.89463
*OsTLP22*	*OsTLP24*	*OsTLP18*	333	15	17	0.13	0.72367
*OsTLP23*	*OsTLP25*	*OsTLP18*	313	20	27	1.04	0.30723
*OsTLP26*	*OsTLP27*	*OsTLP18*	411	34	34	0	1
*SbTLP28*	*SbTLP29*	*SbTLP5*	316	19	30	2.47	0.11608
*SbTLP31*	*SbTLP34*	*SbTLP5*	312	27	32	0.42	0.51508
*SbTLP32*	*SbTLP33*	*SbTLP5*	347	2	2	0	1
*TaTLP1-A*	*TaTLP24-D*	*TaTLP4-D*	483	37	30	0.73	0.39245
*TaTLP1-B*	*TaTLP24-B*	*TaTLP4-D*	486	36	31	0.37	0.5413
*TaTLP12-B*	*TaTLP13-D*	*TaTLP4-D*	321	24	33	1.42	0.23323
*TaTLP15-A*	*TaTLP18-B*	*TaTLP4-D*	391	36	38	0.05	0.81615
*TaTLP15-B2*	*TaTLP18-A*	*TaTLP4-D*	260	28	27	0.02	0.89274
*TaTLP16-A*	*TaTLP17-A*	*TaTLP4-D*	291	36	31	0.37	0.5413
*ZmTLP1*	*ZmTLP35*	*ZmTLP28*	551	36	33	0.13	0.71798
*ZmTLP2*	*ZmTLP34*	*ZmTLP28*	541	34	25	1.37	0.24132
*ZmTLP3*	*ZmTLP22*	*ZmTLP28*	560	11	14	0.36	0.54851
*ZmTLP9*	*ZmTLP12*	*ZmTLP28*	372	6	12	2	0.1573
*ZmTLP13*	*ZmTLP37*	*ZmTLP28*	464	19	22	0.22	0.63941
*ZmTLP16*	*ZmTLP33*	*ZmTLP28*	551	5	7	0.33	0.5637
*ZmTLP20*	*ZmTLP25*	*ZmTLP28*	525	17	23	0.9	0.34278

*Nt, Identical sites in all three sequences; Na, Unique differences in Sequence A; Nb, Unique differences in Sequence B.*

### Gene Architecture, Domain, and Motif Analyses

The number of exons varied from one to three in *O. sativa*, while one to four in *B. distachyon, S. bicolor*, and *T. aestivum*. In the case of *Z. mays*, most of the *TLPs* consisted of one to three exons, while *ZmTLP16, ZmTLP20, ZmTLP25*, and *ZmTLP33* exhibited eleven, seven, eight, and ten exons, respectively. Moreover, a total of 44% (98/222) identified *TLPs* were intronless. Intriguingly, all the *sTLPs* were intronless, except *TaTLP6-A* and *TaTLP20-A1*. The intron phase analysis revealed that the occurrence of a maximum number of introns in phase 1, followed by phase 2, while the least number of introns were in phase 0 (Supplementary Figure 2).

The functional nature of a protein depends upon the occurrence of domain composition. All of the identified cereals’ TLPs consisted of a thaumatin domain (PF00314), which confirmed that they are TLPs. The size of the thaumatin domain ranged from 202–217 AAs to 134–154 AAs in the long and small TLPs, respectively. In addition to the thaumatin domain, ZmTLP16, ZmTLP20, ZmTLP25, and ZmTLP33 also consisted of a nuclear protein 96 (NUP96) domain of ∼211 AAs at the C-terminus of these proteins (Supplementary File 3).

Motif investigation revealed the occurrence of 10 highly conserved motifs in the TLP proteins. Motifs 1–9 were parts of the thaumatin domain, while motif 10 was unknown. Motifs 5, 6, and 8 were the most conserved motifs in TLP proteins, in which the thaumatin signature sequence was present in motif 8 (Supplementary Figure 2). The occurrence of conserved motifs across the cereal species suggested the conserved nature of TLP proteins in related plant species.

### Physicochemical Properties

To understand the various important features, several physicochemical properties of identified TLPs were studied. The TLPs were analyzed for MW, peptide length, isoelectric point (pI), subcellular localization, transmembrane (TM) helix, and signal peptide (Supplementary File 4). The average length of the long and small TLPs ranged from 284–334 AAs to 175–185 AAs in the studied cereal crops, respectively. Similarly, the average MWs ranged from ∼29–35 kDa to ∼17–19 kDa, respectively. However, the average pI ranged from 5.89 to 6.95 and from 4.90 to 6.82 for the long and small TLPs, respectively (Table 3 and Supplementary File 4).

**TABLE 3:** Physicochemical properties of long and small TLP proteins.

Property	Type	*Brachypodium distachyon*	*Oryza sativa*	*Sorghum bicolor*	*Triticum aestivum*	*Zea mays*
**Average protein length**	Long TLPs	299	289	294	284	334
	Small TLPs	185	178	178	177	175
**Average molecular weight (kDa)**	Long TLPs	30.8	29.8	30.0	29.1	35.0
	Small TLPs	19.5	18.1	18.3	18.22	17.7
**Average pI**	Long TLPs	6.18	6.95	6.05	6.18	5.89
	Small TLPs	6.82	6.06	5.74	6.17	4.90

The majority of TLP proteins lacked TM helices, which suggested their soluble/cytoplasmic nature. However, five TLPs of *B. distachyon* and *O. sativa*, seven of *T. aestivum* and *Z. mays*, and 10 TLPs of *S. bicolor* consisted of a single TM region, and OsTLP18, TaTLP4-D, and TaTLP5-D comprised of two TM helices, which suggested their membrane-bound nature (Supplementary File 4).

**FIGURE 4:**
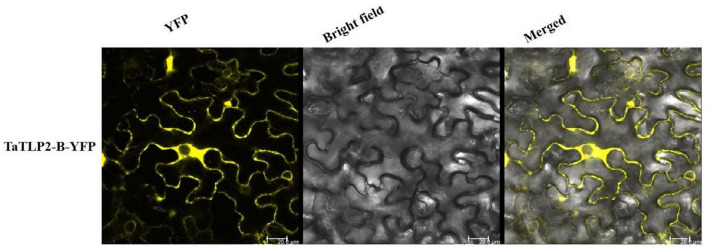
Subcellular localization analysis of TaTLP2-B by YFP fusion. The YFP fluorescence depicts the localization of TaTLP2-B protein majorly in the extracellular region.

A total of 23, 21, 36, 86, and 33 TLPs of *B. distachyon, O. sativa, S. bicolor*, *T. aestivum*, and *Z. mays* consisted of the N-terminal signal peptide, respectively. Further, the majority of TLP proteins were predicted to be localized in the extracellular region, while three TLPs from *O. sativa* (OsTLP10, OsTLP15 and OsTLP16) showed nuclear, and BdTLP16, TaTLP21-A, ZmTLP20, ZmTLP25, and ZmTLP33 showed plasma membrane localization (Supplementary File 4). To validate the subcellular localization, a *TLP* gene (*TaTLP2-B*) was cloned and expressed with YFP as a translational fusion protein. Analysis of YFP fluorescence of fusion protein confirmed its extracellular localization (Figure 4). Moreover, we could also see some fluorescence in the cytoplasmic region. The results suggested that the majority of TaTLP2-B was localized in the extracellular region, where it could be involved in various defense-related functions (such as anti-fungal etc.). The cytoplasmic localization could be due to its translation in the cytoplasm, or it might also function inside the cytoplasm.

### Promoter Region Analysis

Promoter elements are necessary for the regulation of gene expression under different conditions. Therefore, we performed the *cis*-regulatory analyses of *TLPs* to foresee their regulatory mechanisms. Based on their putative functions, the identified *cis*-regulatory elements were segregated into four groups; growth and development, light, hormone, and stress-responsive elements (Supplementary Table 1). The promoter regions of *TLP* genes comprised of several growth-related elements including TE2F2NTPCNA, EBOX, RYREPEAT4, RAV1AAT, POLLEN1LEAAT52, and so on. In the case of light-responsive elements, GT1CONSENSUS, GATA box, GBOXLERBCS, BOXII, and IBOXCORE were some common *cis*-regulatory elements. However, under the hormonal responsive category, DPBFCOREDCD3, ARFAT, ARR1, ERELEE4, and GARE10OSREP1 were abscisic acid, auxin, cytokinin, ethylene, and gibberellic acid-responsive elements, respectively.

### Expression Profiling of the Thaumatin-Like Protein Genes Under Different Tissues and Developmental Stages

**FIGURE 5:**
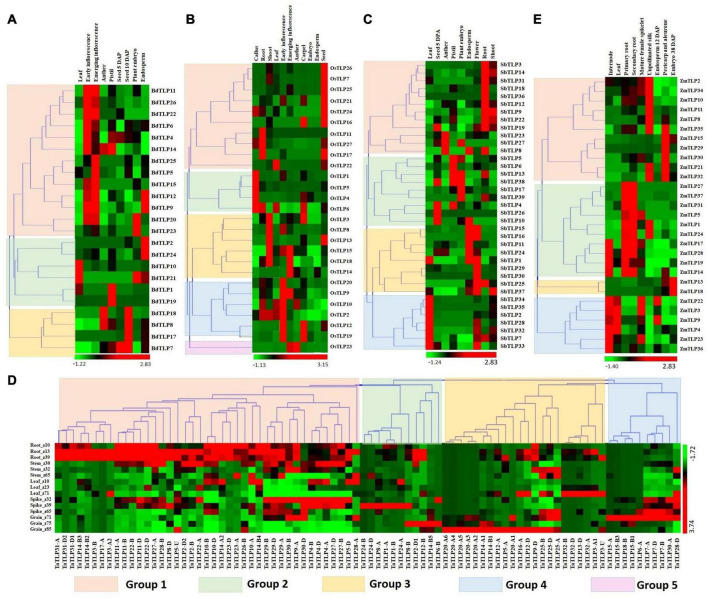
Expression analysis of *TLP* genes of *B. distachyon* (*BdTLP*), *O. sativa* (*OsTLP*). *S. bicolor* (*SbTLP*), *T. aestivum* (*TaTLP*), and *Z. mays* (*ZmTLP*) under various tissue and developmental stages. Heat maps **(A–E)** show the clustering and expression profiling of *TLP* genes in **(A)** *B. distachyon*, **(B)** *O. sativa*, **(C)** *S. bicolor*, **(D)** *T. aestivum*, and **(E)** *Z. mays*. The different clusters are formed based on expression values of *TLP* genes in the respective crop, which are represented with different colors. The color bar depicts the upregulated and downregulated expression with red and green colors, respectively.

Expression analysis of genes is a significant way to understand their involvement in various developmental and physiological processes. Previously, *TLPs* are reported to be involved in plant growth and developmental processes (Munis et al., 2010; Li Z. et al., 2020). Therefore, we performed the expression analysis of *TLPs* under various tissues and their developmental stages using the high-throughput RNA-seq data retrieved from the URGI database and Expression ATLAS (Choulet et al., 2014; Pingault et al., 2015; Papatheodorou et al., 2018). A total of 23, 27, 37, 84, and 29 *TLP* genes showed expression in one or more tissue developmental stages in *B. distachyon, O. sativa, S. bicolor, T. aestivum*, and *Z. mays*, respectively (Figures 5A–E). Based on expression profile, these genes were clustered into 3–5 groups in various crop species. *BdTLPs* of group 1 were highly expressed in early and emerging inflorescence stages, while *BdTLP9* and *BdTLP12*, and *BdTLP20* and *BdTLP23* were upregulated in endosperm and embryo, respectively. Group 2 genes were highly expressed in the leaf, pistil, embryo, and endosperm tissues. However, group 3 genes were highly expressed at 10 days after pollination (DAP) of seeds; moreover, *BdTLP8* and *BdTLP18* were upregulated in the pistil, as well (Figure 5A). In *O. sativa*, the majority of group 1 genes were highly expressed in seed, group 2 in callus, group 3 genes in the shoot, group 4 in inflorescence, and group 5 genes in emerging inflorescence and anther (Figure 5B). In *S. bicolor*, group 1 and group 4 genes showed higher expression in root and leaf tissues, respectively. However, groups 2 and 3 *TLP* genes were highly expressed in various reproductive tissues like anther, pistil, endosperm, embryo, and flower (Figure 5C).

In *T. aestivum*, the majority of group 1 genes exhibited higher expression in root tissue followed by the shoot. However, most of the groups 2, 3, and 4 *TLP* genes were highly expressed in various developmental stages of spike and grain tissues (Figure 5D).

In the case of *Z. mays*, group 1 genes showed significant expression in unpollinated silk, pericarp, and aleurone layer. While the majority of group 2 genes were specifically upregulated in the root tissues, and group 4 genes in the leaf and internode tissues. Group 3 genes were specifically upregulated in the embryo (Figure 5E). These results suggested the role of *TLP* genes in both vegetative and reproductive tissues development in all the studied cereal crops. However, the higher expression of a few genes in a specific tissue or developmental stages suggested their precise role in particular tissue development.

### Expression Profiling of the *TaTLP* Genes Under Biotic and Abiotic Stress Conditions

**FIGURE 6:**
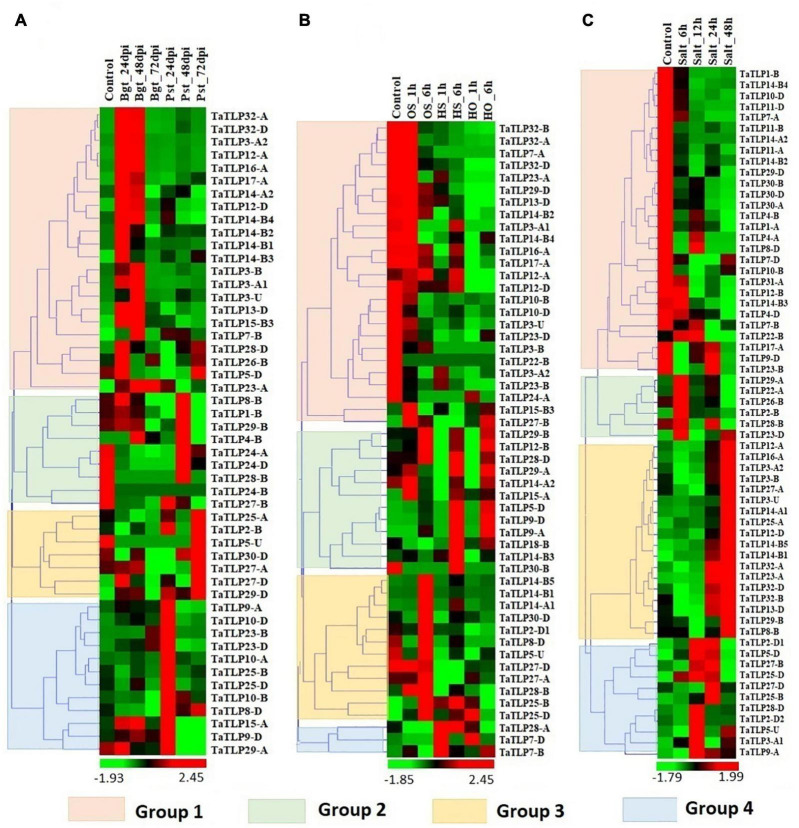
Expression profiling of *TLP* genes of *T. aestivum* under biotic and abiotic stress conditions. Heat maps **(A–C**) show the clustering and expression profiling of *TaTLP* genes under **(A)** *Blumeria graminis* (Bgt) and *Puccinia striiformis* (Pst), **(B)** heat (HS), osmotic (OS), and combined heat and osmotic (HO), and **(C)** salt stress conditions. The clustering of *TaTLP* genes in each heat map is based on their expression pattern in respective stress conditions. The color bar shows high and low expressions with red and green colors, respectively.

Since TLPs belong to the defense-related protein family and are well known for their stress-responsive behavior. Therefore, the expression analysis of *TaTLP* genes was also performed under various biotic and abiotic stress conditions to reveal their roles in stress resistance (Zhang et al., 2014, 2016; Liu et al., 2015). For biotic stress, the available RNA-seq data generated at 24, 48, and 72 h of infestation of two important fungal pathogens, namely, Bgt and Pst were used for expression analysis (Zhang et al., 2014). In *T. aestivum*, a total of 50 *TaTLP* genes exhibited differential expression (≥ 2 fold) in these stress conditions, which could be clustered into four groups (Figure 6A). The majority of group 1 *TaTLP* genes were upregulated at 24 and 48 h of Bgt infestation. However, all the group 3 and 4 *TaTLP* genes were significantly upregulated at the late (72 h) and early (24 h) periods of Pst infestations, respectively. *TaTLP3-A2*, *TaTLP12-A*, and *TaTLP32-A* were the most upregulated genes after Bgt infection with 37, 36, and 35 folds, respectively. However, in the case of Pst infestation, *TaTLP10-A* (51 fold up) and *TaTLP27-D* (17 fold up) were highly upregulated.

Abiotic stresses are another major threat to plants, which affect various physiological and biological processes. To understand the putative role of *TaTLPs* under abiotic stress conditions, expression analysis was carried out under osmotic (OS), heat (HS), and combined heat and osmotic (HO) stresses using online available RNA-seq data (Liu et al., 2015). A total of 52 *TaTLPs* exhibited significant differential expression in these stresses, which formed four clusters in the heat map (Figure 6B). Almost all the genes of group 1 were downregulated in the OS, HS, and HO stresses, except *TaTLP12-A* that was slightly upregulated in OS treatment. In the case of group 2, the majority of genes were upregulated at 6 h of OS, HS, and HO treatments, while either unaffected or downregulated at 1 h of treatment. These might be the late responsive *TaTLP* genes. Most of the group 3 and group 4 *TaTLP* genes were found to be OS and HS responsive, respectively.

Expression analysis of the *TaTLP* genes was also performed at 6, 12, 24, and 48 h of salt stress using available RNA-seq data (Zhang et al., 2016). Out of 93 genes, 63 *TaTLP* genes were found to be differentially expressed under salt stress, which were clustered into 4 groups, based on their expression patterns (Figure 6C). All the group 1 genes were downregulated at all the stages of salt stress treatment. In contrast, all the group 3 genes were found significantly upregulated at 48 h of treatment. However, group 2 genes were upregulated at the early stage (6 h) of treatment, while they get normalized at later stages. The results suggested that the group 2 and group 3 genes are early and late responsive *TaTLP* genes, respectively.

**FIGURE 7:**
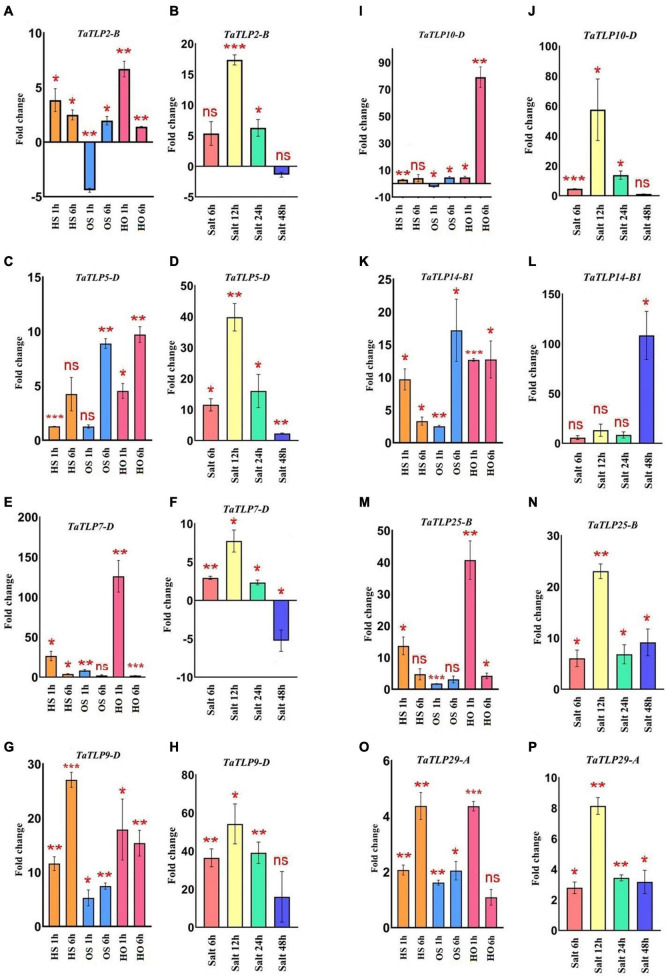
qRT-PCR analysis of eight *TaTLP* genes. Expression patterns in the form of fold changes have been shown in the bar graphs. **(A,C,E,G,I,K,M,O)** Show expression of *TaTLP2-B*, *TaTLP5-D, TaTLP7-D, TaTLP9-D, TaTLP10-D, TaTLP14-B1, TaTLP25-B, and TaTLP29-A* genes, under HS, OS, and HO stress conditions, while **(B,D,F,H,J,L,N,P)** shows the expression pattern of these genes under salt stress, respectively. Significance between the control and treated conditions is carried using a two-tailed student’s *t*-test. The ns, *, **, and *** markings represent the significance at *p*-value > 0.05, ≤0.05, ≤0.01, ≤0.001, respectively.

In addition to *in silico* analysis, qRT-PCR analyses of eight *TaTLP* genes were performed using the gene-specific primers at similar heat, osmotic, and salt stress treatments for the validation of expression profile (Figures 7A–P and Supplementary File 5). Overall, the result was in agreement with the expression observed using RNA-seq data, with a few exceptions. In the case of heat stress, *TaTLP2-B*, *TaTLP7-D*, *TaTLP14-B1*, and *TaTLP25-B* were more upregulated at 1 h, while other genes were highly upregulated at 6 h of treatment. In the case of osmotic stress, *TaTLP-7D* was exclusively more upregulated at 1 h, whereas other genes were upregulated at 6 h. However, *TaTLP2-B* and *TaTLP10-D* were downregulated at OS 1 h. The majority of genes were highly upregulated at 1 h HO treatment. In the case of salt stress, seven genes were highly upregulated at 12 h of salt stress, except *TaTLP14-B1* that showed maximum expression at 48 h. The results further confirmed that a few *TaTLP* genes are early while others are late responsive.

### Comparative Expression Profiling of the *TaTLP* Paralogous Genes

**FIGURE 8:**
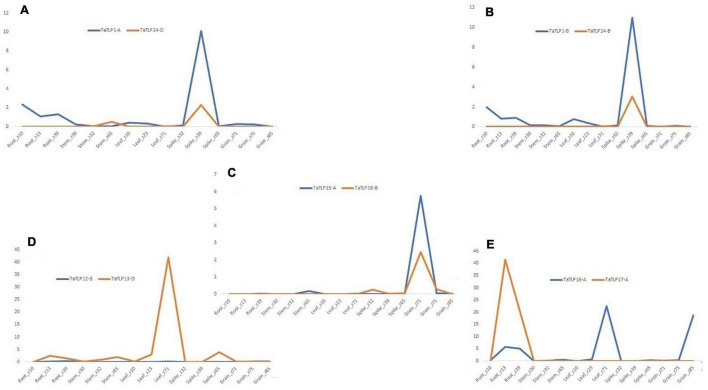
Comparative expression analysis of duplicated gene pairs in *T. aestivum*. **(A–E)** Represent the comparative expression pattern of paralogous *TaTLPs* under various tissue and developmental stages. Based on their expression trend, *TaTLPs* are categorized into the retention of function **(A–C)** and pseudo functionalization types **(D,E)**.

To understand the function of duplicated genes, a comparative expression profiling of each paralogous pair was performed. Generally, based on expression pattern, duplicated genes could categorize into the retention of function, pseudo-functionalization, and neo-functionalization. Out of the six paralogous gene pairs, three pairs (*TaTLP1-A-TaTLP24-D, TaTLP1-B-TaTLP24-B*, and *TaTLP15-A-TaTLP18-B*) showed a similar trend of expression, which suggested retention of function in these duplicated genes (Figures 8A–C). Two duplicated pairs (*TaTLP12-D-TaTLP13-D* and *TaTLP16-A-TaTLP17-A*) showed insignificant expression of one of the gene suggested pseudo-functionalization (Figures 8D,E). Retention of function in the majority of duplicated genes and the absence of neo-functionalization suggested functional conservation in *TLP* genes during evolution.

### Cloning and Functional Characterization of the *TaTLP2-B* in *Saccharomyces cerevisiae*

**FIGURE 9:**
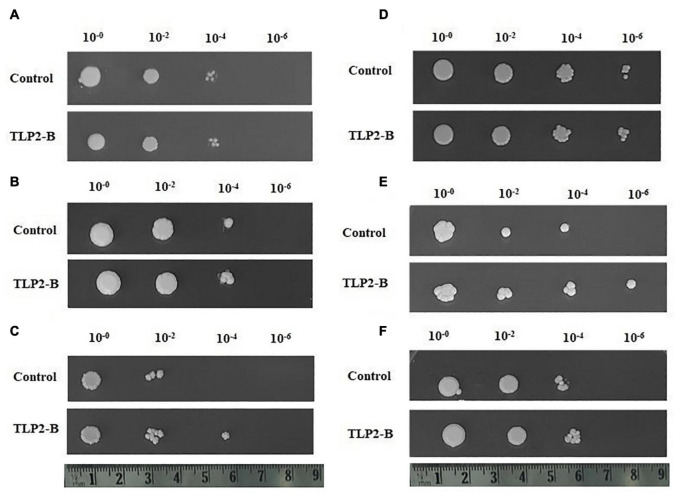
Spot assay of recombinant *Saccharomyces cerevisiae* (yeast) cells under various stress conditions. **(A)** Shows similar growth of both TaTLP2-B (pYES2.1-TaTLP2-B) and control vector (pYES2.1/V5-His/lacZ) containing yeast cells under control conditions. **(B–F)** Shows the spot assay analysis of *TaTLP2-B* and control vector containing yeast cells in **(B)** cold (4°C), **(C)** osmotic (30% PEG), **(D)** heat (37°C), **(E)** combined heat and osmotic (37°C and 30% PEG), and **(F)** salt (1M NaCl) stress conditions, respectively.

In our study, since the *TaTLP2-B* exhibited significant differential expression under abiotic stress conditions, it was selected for functional characterization. *TaTLP2-B* ORF was amplified using end primers and cloned into the pYES2.1 yeast expression vector. The *LacZ* gene-containing vector was used as a control (Figures 9A–F). In the spot assay, we observed similar growth of both control (LacZ) and TaTLP2-B expressing yeast cells in the controlled conditions (Figure 9A). Since the yeast optimum growth temperature is 30°C, we used 37 and 40°C for heat stress treatment. However, we observed higher growth inhibition at 40°C treatment, therefore, 37°C treatment was used for further analysis. In the case of 20% PEG, we could not see significant difference at the lower dilutions, therefore 30% PEG was used for further analysis. Similarly, less than 1 M NaCl could not cause significant inhibition at lower dilutions. The results revealed that in the case of cold (4°C), osmotic (30% PEG), heat, combined heat and osmotic (37°C and 30% PEG), and salt (1M NaCl) stress conditions, TaTLP2-B recombinant cells exhibited higher growth than the control cells (Figures 9B–F). The results suggested that the overexpression of the TaTLP2-B provided abiotic stress tolerance to the recombinant yeast cells. This gene can be used for the development of abiotic stress-resistant transgenic crops in future studies.

## Discussion

Thaumatin-like proteins are important proteins involved in the tissue development and stress resistance pathways of plants (Liu et al., 2010a). Therefore, we have studied the TLPs in five major cereals including *B*. *distachyon*, *O*. *sativa*, *S*. *bicolor*, *T*. *aestivum*, and *Z*. *mays*. In the present study, we have identified a total of 26, 27, 39, 93, and 37 *TLPs* in the genome of *B*. *distachyon*, *O*. *sativa*, *S*. *bicolor*, *T*. *aestivum* and *Z*. *mays*, respectively. Variable numbers of the *TLP* genes in different plants have been reported in earlier studies. For instance, 24, 33, and 49 *TLP* genes have been reported in diploid *A*. *thaliana*, *Vitis vinifera*, and *Populus trichocarpa*, respectively (Cao et al., 2016; Yan et al., 2017). In the case of *O*. *sativa* and *Z*. *mays*, 49 *TLP* genes in each are reported in the earlier study (Cao et al., 2016), because probably they had not removed the genes with incomplete thaumatin signature motifs, as done in the present study. The occurrence of the highest number of *TLP* genes in *T*. *aestivum* could be attributed to their complex allohexaploid genome configuration (AABBDD) (Sharma A. et al., 2020). Further, differential expansion of the *TLP* genes in various plant species might be attributed to the number of DEs.

The duplication events are responsible for the expansion of gene families and these are the major forces to attain neo-functionality by causing genetic variability (Appels et al., 2018). In DEs analysis, our results suggested that tandem DEs are a major factor in *B*. *distachyon*, *O*. *sativa*, and *S*. *bicolor* and segmental DEs in *T*. *aestivum* and *Z*. *mays* behind the gene expansion of TLP family. The role of tandem and segmental duplications in the evolution of TLP gene family has been earlier reported in various plant species including *A*. *thaliana*, *O*. *sativa*, and *Z*. *mays* (Cao et al., 2016). The higher number of paralogous genes in *T*. *aestivum* and *Z*. *mays* might be attributed to their larger genome size and the presence of more transposable elements (Gaut, 2002; Ellegren, 2008).

Furthermore, the divergence time of monocots from eudicots was estimated as ∼170–235 Mya, which were further diverged into grasses around ∼77 Mya (Yang et al., 1999; Gaut, 2002). Therefore, the paralogous *TLP* genes in *B*. *distachyon* might have evolved before the divergence of grasses. The main duplication incidence in *S*. *bicolor* and *O*. *sativa* genome was estimated at around ∼70 Mya (Paterson et al., 2004). However, our results indicated that the majority of paralogous *TLP* genes of *O*. *sativa* and *S*. *bicolor* are evolved after the main DE occurrence. In *Z*. *mays*, paralogous *TLP* genes were probably formed close to the divergence time of maize and sorghum, i.e., ∼11–28 Mya (Paterson et al., 2004). However, in the case of *T*. *aestivum*, paralogous *TLP* genes were probably evolved earlier than the hybridization event of A, B, and D subgenomes (Marcussen et al., 2014). However, two duplicated pairs, having NUP 96 domain, showed their recent incidence of DEs, which showed that TLPs might be acquiring the new functions over the course of evolution. Duplicated genes face various environmental forces and natural pressures during the process of evolution (Ellegren, 2008). Our results suggested the purifying selection of *TLP* genes as a major force of selection, as reported in the various other plant species (Cao et al., 2016; Liu et al., 2020). Moreover, positive selection has also been reported for *TLP* genes in poplar, which suggested the role of varied natural selection procedures during TLPs’ evolution (Liu et al., 2010b). Since the TLPs are paraphyletic in origin, they are supposed to be derived from multiple ancestral genes (Shatters et al., 2006). Clustering of identified TLPs in multiple clades is in agreement with the paraphyletic nature of origin. Further, a variable number of TLPs in each clade is due to the differential duplication of *TLP* genes. Similarly, the uneven distribution and paraphyletic nature of TLPs have also been reported by previous studies (Cao et al., 2016; Liu et al., 2020). The tight clustering of homeologous and paralogous genes could be due to the high sequence homology among the respective sequences.

The organization of introns and exons of *TLPs* have been reported to be in the range of one to ten exons (Liu et al., 2010a; Petre et al., 2011; Cao et al., 2016). On similar patterns, our results suggest comparable findings with one to four exons in most of the TLPs. However, most of the monocots’ TLPs have intron-less nature, which was also found in our analysis (Cao et al., 2016). Additionally, our results with respect to the protein lengths, MWs, and isoelectric points of long and small TLP proteins were found in accordance with previously studied TLPs (Liu et al., 2010a; Petre et al., 2011; Cao et al., 2016). In our analysis, the TaTLP2-B was found to be localized in the extracellular region. Similarly, the extracellular localization of a wheat TLP (TaLr19TLP1) is also reported in an earlier study (Zhang et al., 2018).

In addition, the presence of thaumatin-like domain in all the TLP proteins, make our studies similar to previously reported findings. However, four ZmTLPs (ZmTLP16, ZmTLP20, ZmTLP25, and ZmTLP33) have an additional NUP 96 domain. The NUP 96 domain is reported to be involved in plant development and stress resistance against pathogens (Zhang and Li, 2005). Various abiotic and biotic stress-responsive elements were also found in most of the studied *TLPs*, for instance, DRE2COREZMRAB17, ASF1, BIHD1, CBFHV, CGG-box, GCCCORE, LTRECOREATCOR15, MYB, MYC, WRKY1, W-box, T/G-box, and so on. Various plant hormone-related and stress-related *cis*-regulatory elements have also been reported in four cotton species (Li Z. et al., 2020). Moreover, in previous studies, the elements like ERE, W-box, and WRKY were found to be involved in plant growth and stress resistance (Gou et al., 2007; Hiroyuki and Terauchi, 2008; Rushton et al., 2010). The occurrence of numerous *cis*-regulatory elements suggested diverse functions of *TLP* genes in plants. Further, the expression profiling of *TLPs* under various tissues and developmental stages also advocated the putative involvement of these genes in the development of vegetative and reproductive tissues. Similar expression trend in various plant tissues and their role in reproductive tissues like flowers and seeds has also been reported in earlier studies; for instance, *TLP* genes in *Gossypium hirsutum* also showed their varied expression pattern in vegetative as well as in reproductive organs (Fils-Lycaon et al., 1996; Seo et al., 2008; Li Z. et al., 2020).

The role of *TLP* genes in fungal resistance has been reported in earlier studies against numerous pathogens including *Bipolaris sorokiniana* (Cui et al., 2021), *Fusarium* sp. (Mackintosh et al., 2007), *Microdochium nivale* (Kuwabara et al., 2002), *Puccinia. Triticina* (Cui et al., 2021), *Rhizoctonia solani* (Naseri et al., 2012), and *Verticillium dahlia* (Li Z. et al., 2020) in wheat and other plant species. Bioassay of *Plasmopara viticola* in transgenic *V. vinifera* showed that the overexpression of *VaTLP* gene helped in the reduction of hyphe growth (He et al., 2017). The exact mechanism of TLPs is ambiguous; however, in the oomycetes fungus, PR5 are reported to degrade the β-1,3-glucans (Abad et al., 1996; Grenier et al., 1999). Moreover, a recent study also showed the interaction of *TaTLP1* with *PR1* genes of *T. aestivum* in two-hybrid experiments (Y2H) (Wang et al., 2020). The differential expression of *TaTLP* genes in response to Pst and Bgt infestation further established their roles in fungal stress responses.

The qRT-PCR of a few selected genes was carried out to validate the expression data obtained from the RNA-seq analyses. The *TaARF* gene was used as an internal control as reported in earlier studies (Shumayla et al., 2019; Tyagi et al., 2021). Further, the RNA-seq data analysis revealed invariable expression of *TaARF* gene in all the tissue developmental stages and stress conditions, therefore it was used as a reference gene in our qRT-PCR analyses. The qRT-PCR results showed the variable expression of *TaTLPs* at early and late stages of heat, osmotic, and combined heat and osmotic and salt stress treatments. The expression trends were similar as observed in RNA-seq data. For instance, *TaTLP5-D* exhibited the maximum expression at 6 h of osmotic and combined heat and osmotic stresses, and 12 h of salt stress in both qRT-PCR and *in silico* expression analyses. Similarly, in the case of *TaTLP14-B1* and *TaTLP29-A*, the maximum upregulation was seen at the later stages of osmotic and salt stresses in both the RNA-seq data and qRT-PCR results. These results supported the consistency in the expression data of RNA-seq and qRT-PCR, and suggested the stress-responsive nature of *TaTLP* genes. Further, the *TaTLP2-B* exhibited significant differential expression in various stress conditions, therefore it was used for cloning and functional characterization in yeast. Moreover, similar to our *in silico* and qRT-PCR finding’s differential expression, upregulation of *TLP* genes at various hours of osmotic, salt, and cold stress treatment has also been reported in other plants like *Brassica rapa* and *Gossypium* sp. (Ahmed et al., 2013; Li Z. et al., 2020). Seven genes of *B. rapa* (*BrTLP1, 2, 3, 8, 12, 13, and 20*) exhibited differential expression at the different time intervals of cold, drought, and salt stresses (Ahmed et al., 2013). Moreover, the qRT-PCR experiment showed the upregulation of *BoTLP1* of *Brassica oleracea* L. var. *Italica* after 4, 8, and 24 h of salt (200 mM Nacl) and drought stresses (300 mM mannitol) (He et al., 2021).

The overexpression of *TaTLP2-B* provided significant tolerance against various abiotic stress conditions in yeast cells. Similarly, the overexpression of *TLP* genes provided increased tolerance against various abiotic stresses in tobacco, cotton, Arabidopsis, broccoli, and so on (Rajam et al., 2007; Misra et al., 2016; Li Z. et al., 2020; He et al., 2021). In a recently conducted study, the important role of a cotton *TLP* (*GhTLP19*) gene was studied in combating drought stress (Li Z. et al., 2020). Moreover, the *GbTLP1* of *G*. *barbadense* and *ObTLP1* of *Ocimum basilicum* were found effective against drought and salt stresses in two separate studies (Munis et al., 2010; Misra et al., 2016). The transgenic line of *Brassica oleracea L. var. Italica* with overexpressed *BolTLP1* showed remarkable resistance against drought and salt stresses (He et al., 2021). The abscisic acid (ABA) signaling cascade is a central pathway in the regulation of salt, drought, and other abiotic stresses. In a recent study, thaumatin mutant plants showed alteration in the ABA signaling pathway, which ultimately leads to increased susceptibility of plants to abiotic stresses (Park and Kim, 2021). Collectively, these findings depict that the TLP proteins could be utilized to develop stress resistant transgenic cereal crops, which will be a boost for future agriculture.

## Conclusions

Thaumatin-like proteins, a part of the PR5 protein family, are known to be involved in various biotic and abiotic stresses. The curiosity to unravel the various possibilities led us to a detailed characterization of a total of 222 *TLP* genes in five cereal crops. Phylogenetic analysis revealed the paraphyletic origin of TLPs in cereals, while the occurrence of DEs suggested the role of paralogous genes in the expansion of the TLP gene family. Gene expression analysis using RNA-seq data and qRT-PCR suggested the important roles of *TLP* genes in plant growth and development and abiotic and biotic stress responses. Significant tolerance in the *TaTLP2-B* expressing yeast cells further established their role in stress response. Overall, the study revealed that the *TLP* genes can be used for stress-resistant transgenic crop development. Since the identified genes are directly derived from the food crops, they would be easily deregulated from the regulatory authority of the transgenic crops. Further, the current study will facilitate the detailed functional characterization of *TLP* genes of cereal crops in future studies.

## Data Availability Statement

The datasets presented in this study can be found in online repositories. The names of the repository/repositories and accession number(s) can be found below: URGI, PRJNA243835; NCBI BioProject, PRJNA243835, SRP045409, and SRP062745.

## Author Contributions

SU conceived the idea. AS and SU designed the experiments. AS, HS, and RR performed the experiments. AS, HS, and AP analyzed the data. AS, HS, and SU wrote the manuscript. All authors have read and finalized the manuscript.

## Conflict of Interest

The authors declare that the research was conducted in the absence of any commercial or financial relationships that could be construed as a potential conflict of interest.

## Publisher’s Note

All claims expressed in this article are solely those of the authors and do not necessarily represent those of their affiliated organizations, or those of the publisher, the editors and the reviewers. Any product that may be evaluated in this article, or claim that may be made by its manufacturer, is not guaranteed or endorsed by the publisher.

## Footnotes

1
http://wheat.pw.usda.gov/ggpages/wgc/98/Intro.htm

2
http://plants.ensembl.org/

3
http://www.plantbreeding.wur.nl/uk/software_mapinspect.html.2012

4
https://www.ebi.ac.uk/Tools/msa/clustalo

5
https://urgi.versailles.inra.fr/files/RNASeqWheat/

## Supplementary Material

Supplementary Figure 1 Multiple sequence alignment of 222 Thaumatin-like proteins (TLPs) of *B. distachyon, O. sativa, S. bicolor*, *T. aestivum*, and *Z. mays*. Conserved cysteine residues are marked with an asterisk. The red dot denotes the REDDD motif. The FF hydrophobic motif is highlighted with a green triangle. The thaumatin signature motif, conserved domain, and amino acids forming the bottom of the acidic cleft are marked with sky blue, red, and brown lines, respectively.

Supplementary Figure 2 Intron/exon organization and conserved motifs of TLPs of *B. distachyon*, *O. sativa*, *S. bicolor, T. aestivum*, and *Z. mays*.
