# Supplementary material for: Molecular Characterization Revealed the Role of Thaumatin-Like Proteins of Bread Wheat in Stress Response
Source: Front Plant Sci. 2022 Jan 11;12:807448. doi: 10.3389/fpls.2021.807448 (PMC8786798; doi:10.3389/fpls.2021.807448)
Supplement: Supplementary Figure 1 — Multiple sequence alignment of 222 Thaumatin-like proteins (TLPs) of B. distachyon, O. sativa, S. bicolor, T. aestivum, and Z. mays. Conserved cysteine residues are marked with an asterisk. The red dot denotes the REDDD motif. The FF hydrophobic motif is highlighted with a green triangle. The thaumatin signature motif, conserved domain, and amino acids forming the bottom of the acidic cleft are marked with sky blue, red, and brown lines, respectively. [file Data_Sheet_1.ZIP › Supplimentary files/Supplementary figure 1.pdf]

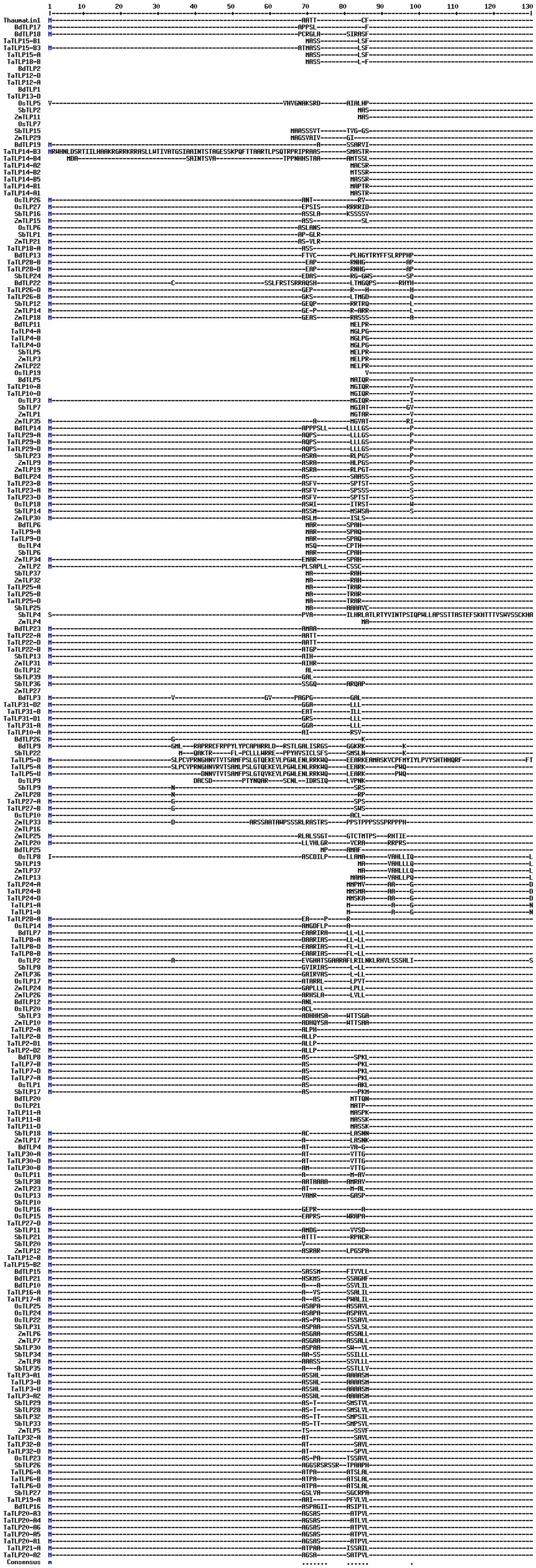

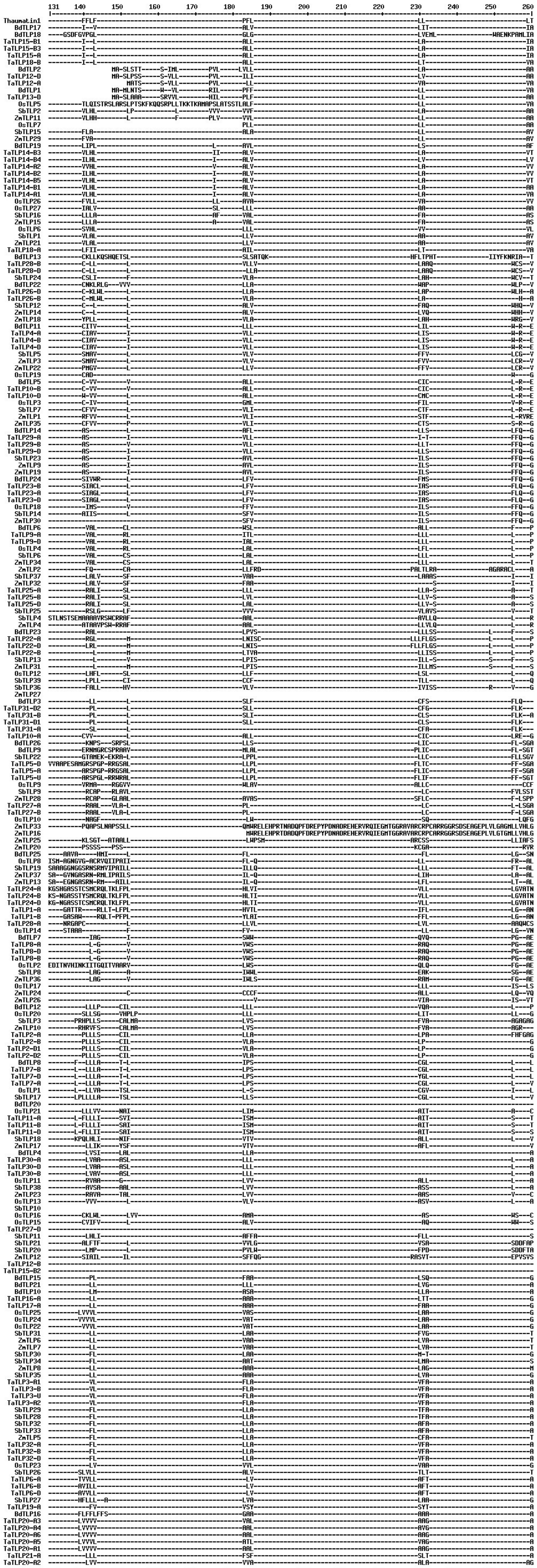

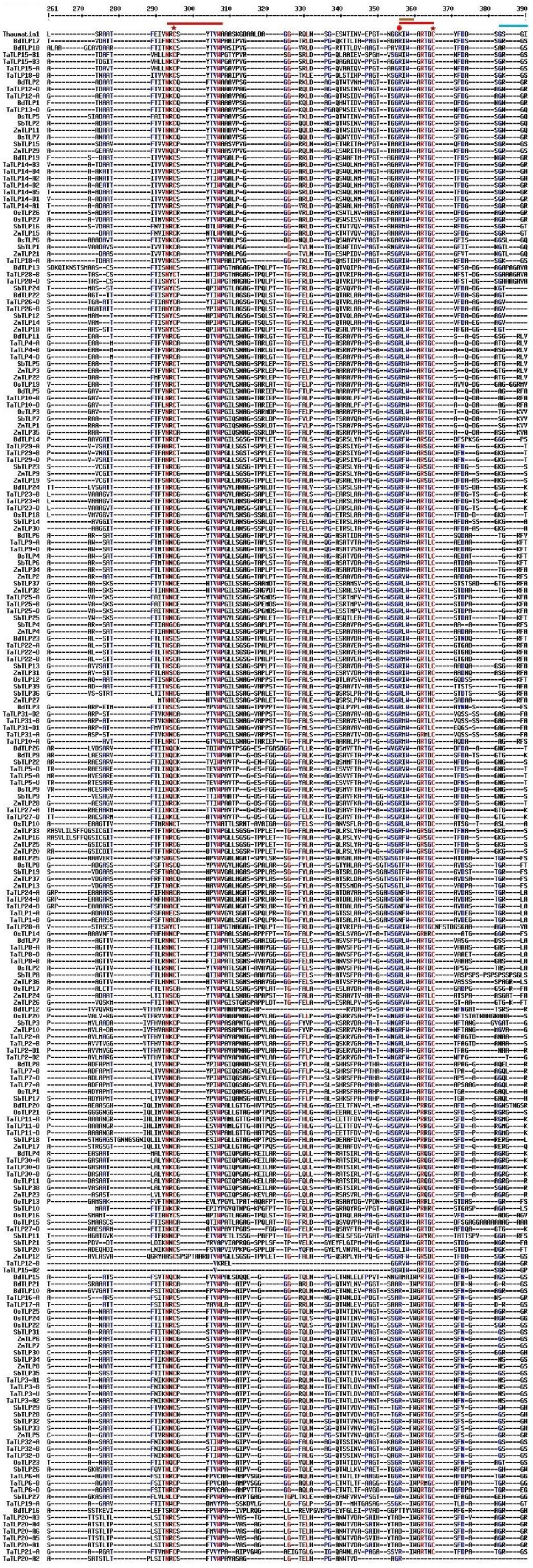
261 270 280 290 300 310 320 330 340 350 360 370 380 390

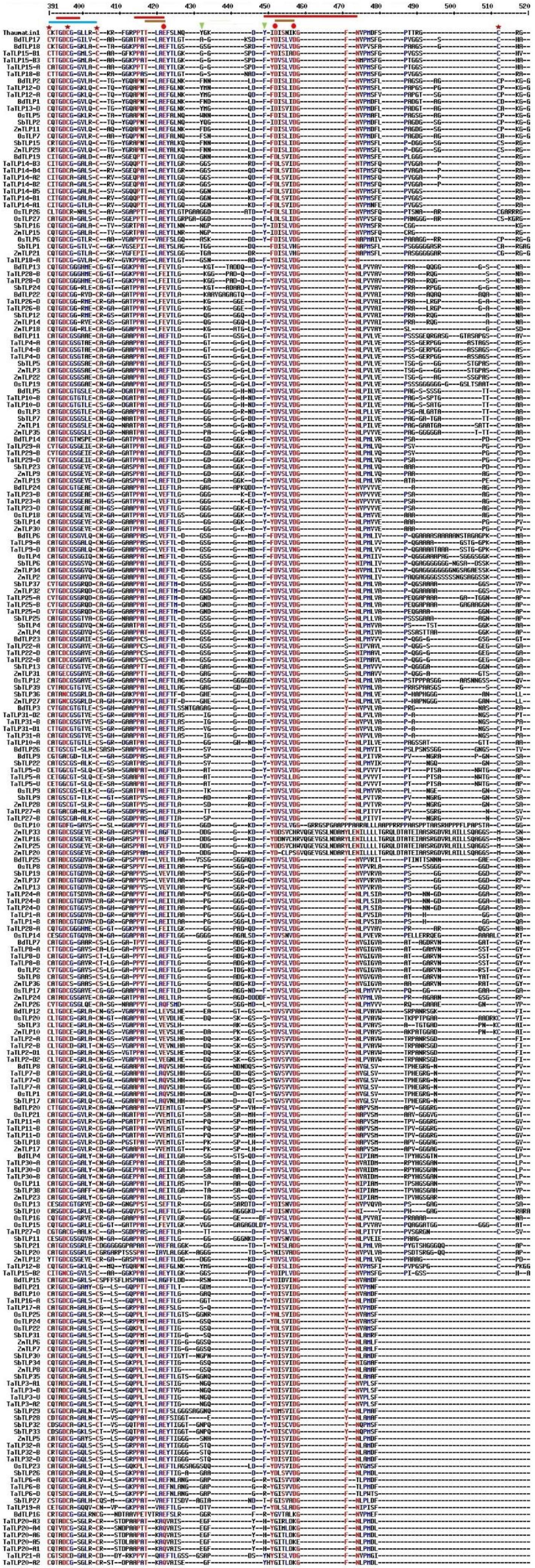
391
400
410
420
430
440
450
460
470
480
490
500
510
520

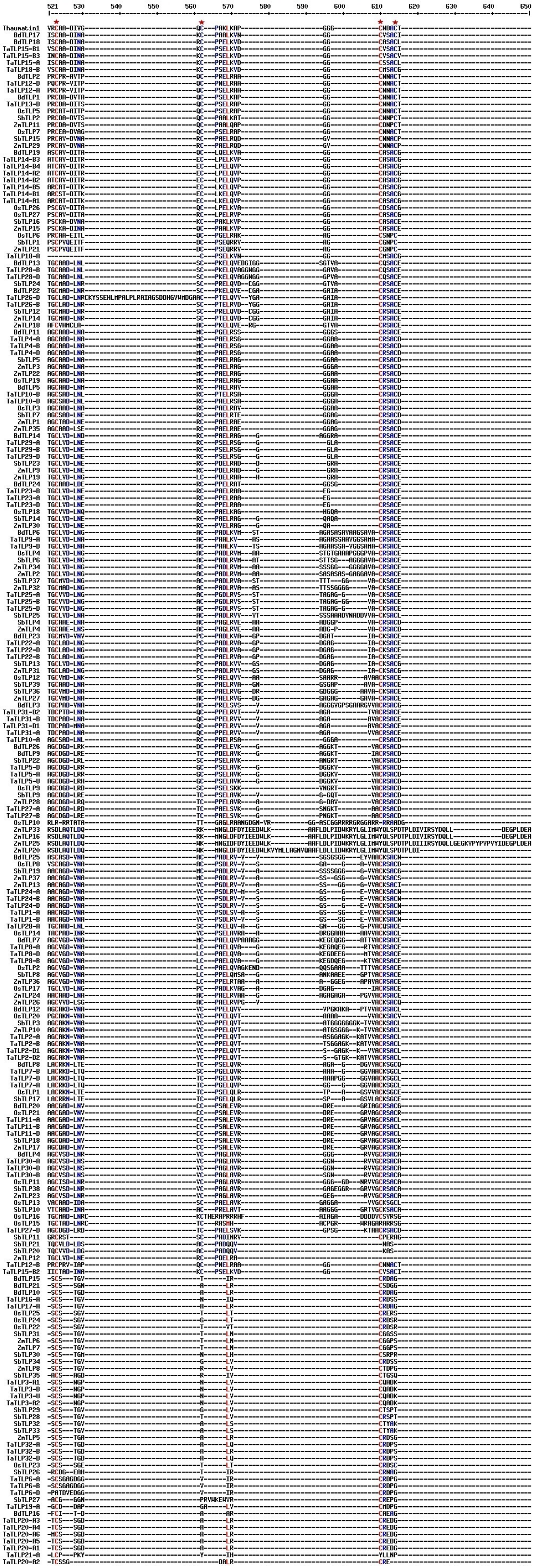
521 530 540 550 560 570 580 590 600 610 620 630 640 650

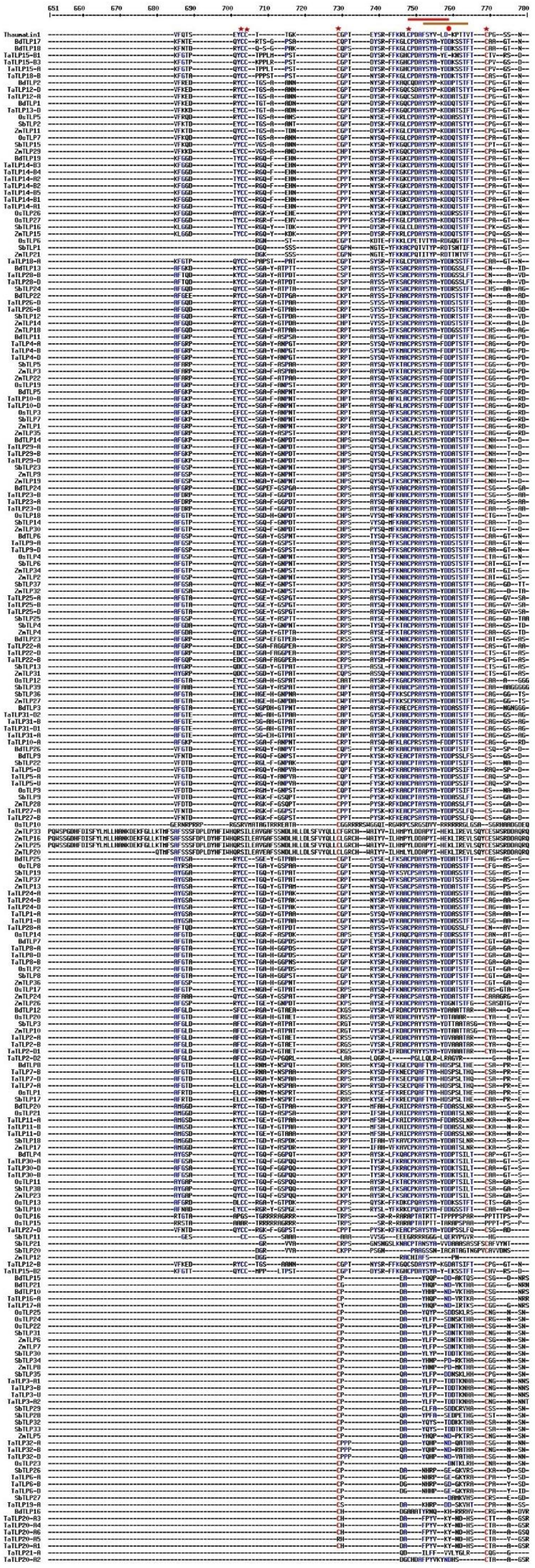
651 660 670 680 690 700 710 720 730 740 750 760 770 780

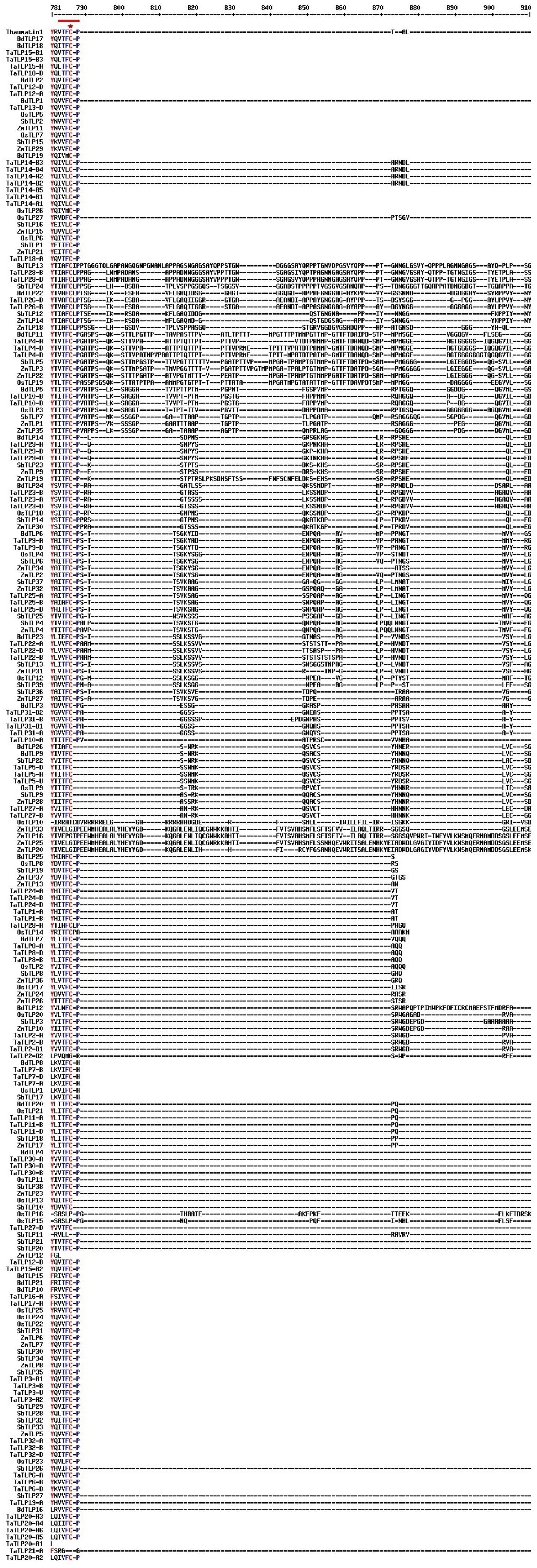
781 790 800 810 820 830 840 850 860 870 880 890 900 910

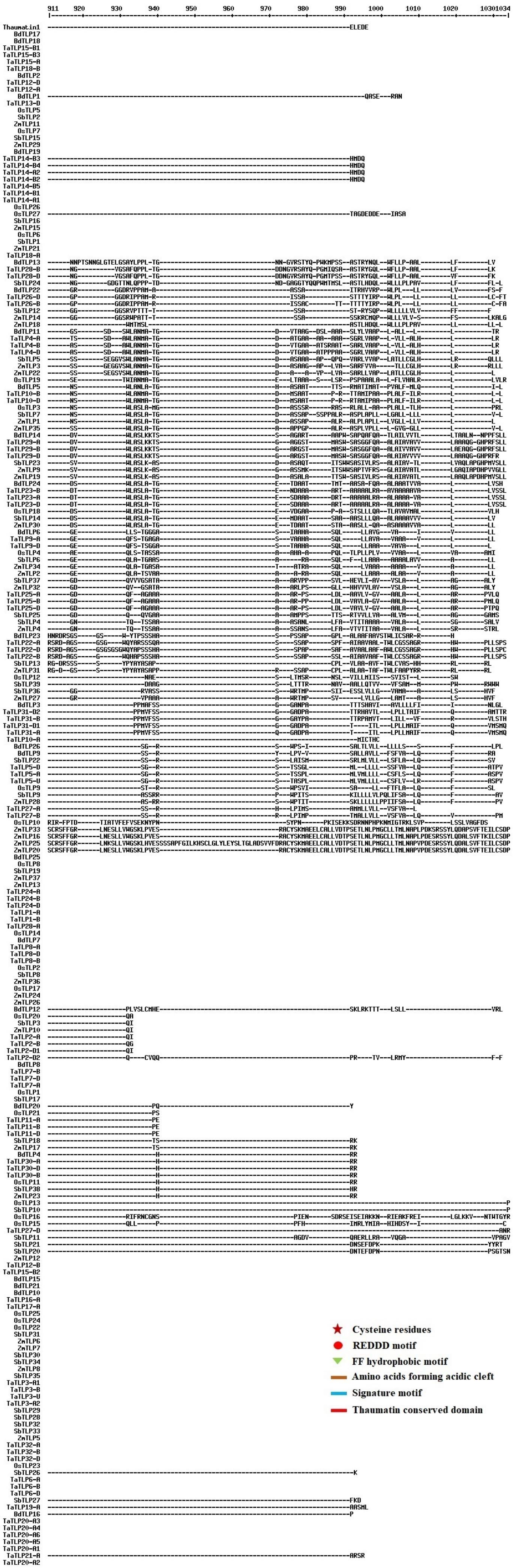
Cysteine residues
REDDD motif
FF hydrophobic motif
Amino acids forming acidic cleft
Signature motif
Thaumatin conserved domain
