# Supplementary figures and images for: Molecular Characterization Revealed the Role of Thaumatin-Like Proteins of Bread Wheat in Stress Response
Source: Front Plant Sci. 2022 Jan 11;12:807448. doi: 10.3389/fpls.2021.807448 (PMC8786798; doi:10.3389/fpls.2021.807448)

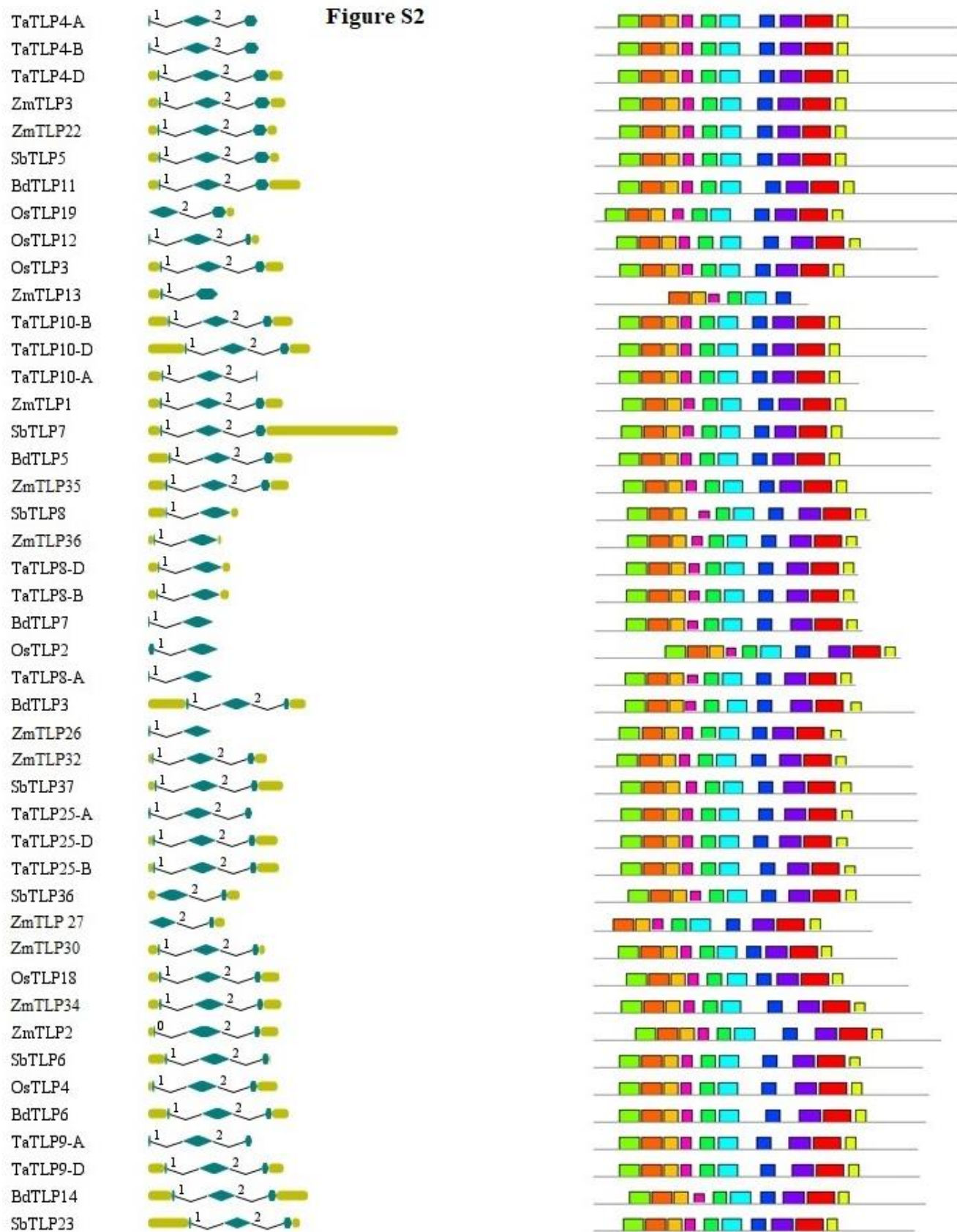

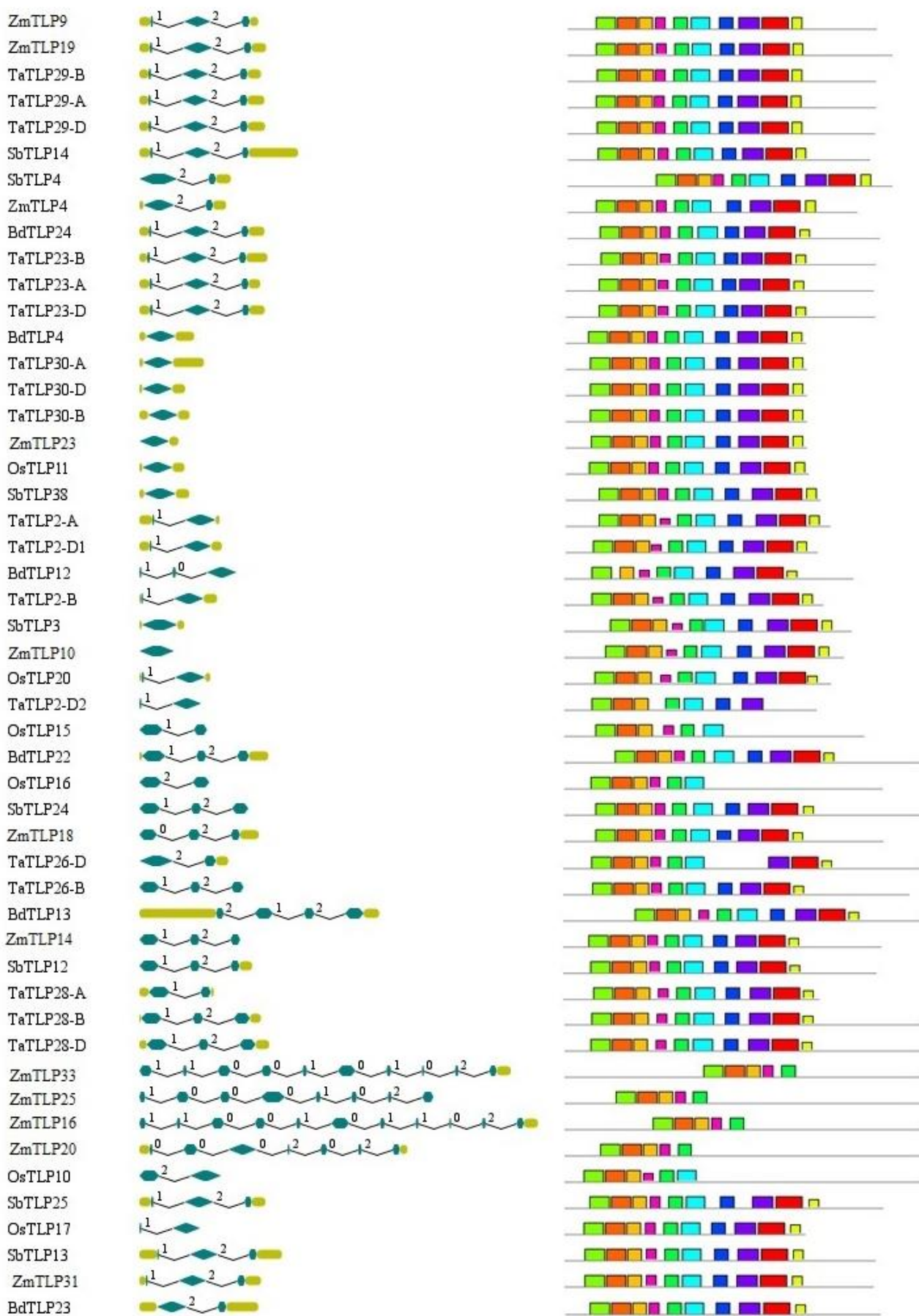



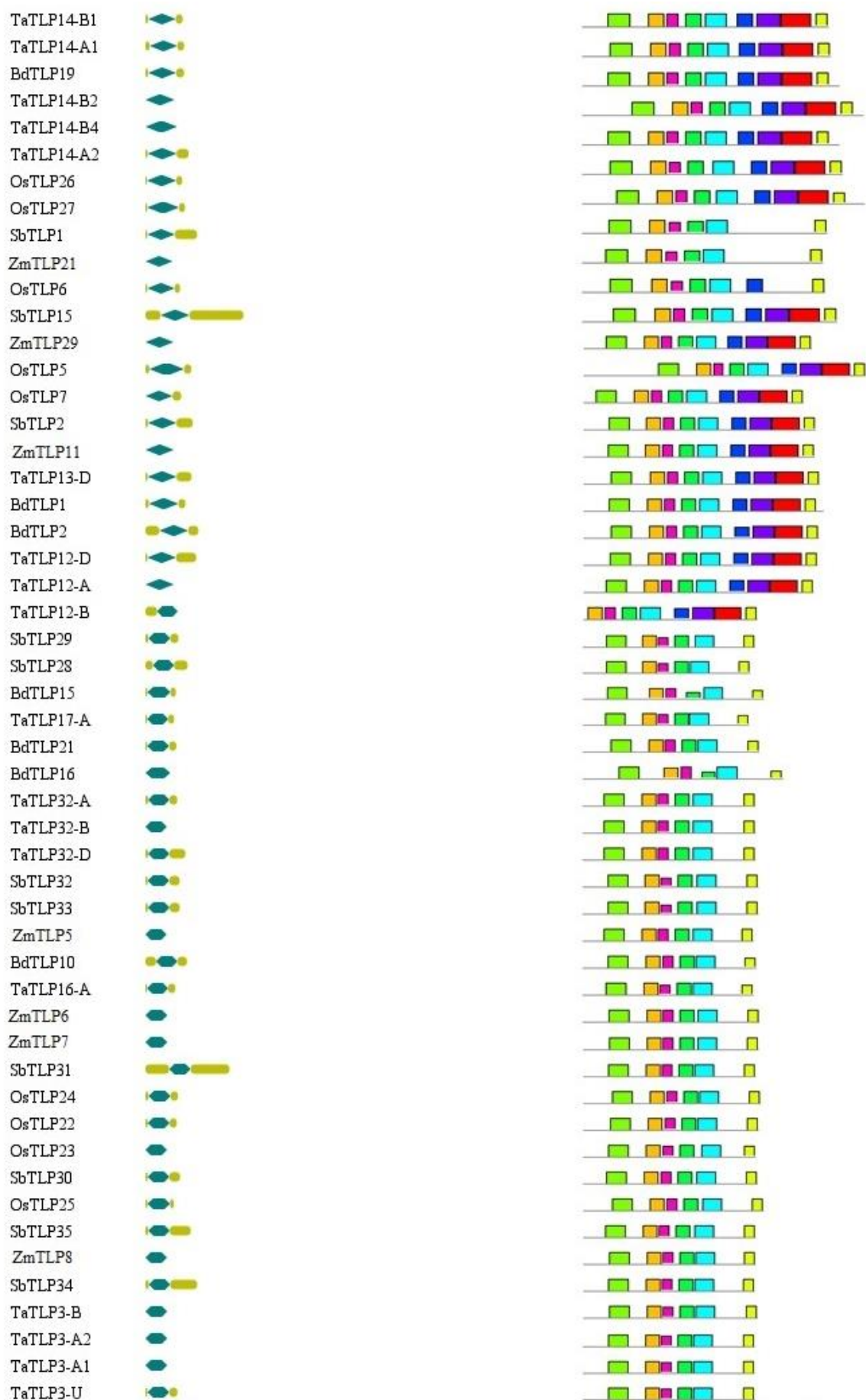

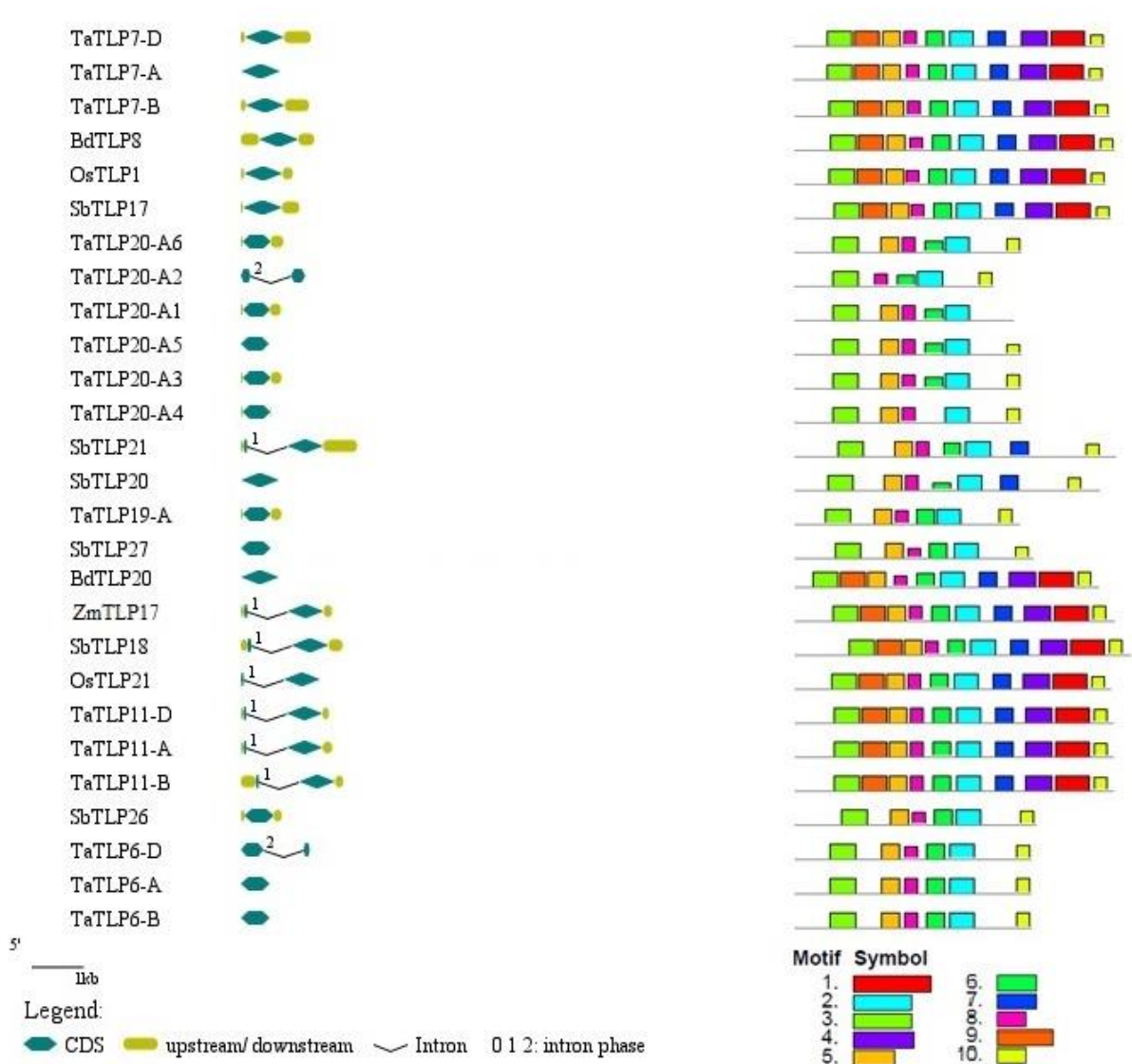

Supplement: Supplementary Figure 1 — Multiple sequence alignment of 222 Thaumatin-like proteins (TLPs) of B. distachyon, O. sativa, S. bicolor, T. aestivum, and Z. mays. Conserved cysteine residues are marked with an asterisk. The red dot denotes the REDDD motif. The FF hydrophobic motif is highlighted with a green triangle. The thaumatin signature motif, conserved domain, and amino acids forming the bottom of the acidic cleft are marked with sky blue, red, and brown lines, respectively. [file Data_Sheet_1.ZIP › Supplimentary files/Supplementary figure 2.pdf]
